# Highly accurate image registration for 3D multiplexed cyclic imaging using dense labeling in expandable tissue gels

**DOI:** 10.1371/journal.pbio.3003240

**Published:** 2025-07-03

**Authors:** Hyunwoo Kim, Joon-Goon Kim, Jueun Sim, Hoyeon Nam, In Cho, Hyejin Shin, Junyoung Kwon, Dae-Hyeon Song, Seoungbin Bae, Young-Gyu Yoon, Taeyun Ku, Jae-Byum Chang

**Affiliations:** 1 Department of Materials Science and Engineering, Korea Advanced Institute of Science and Technology, Daejeon, 34141, Republic of Korea; 2 Graduate School of Medical Science and Engineering, Korea Advanced Institute of Science and Technology, Daejeon, 34141, Republic of Korea; 3 School of Electrical Engineering, Korea Advanced Institute of Science and Technology, Daejeon, 34141, Republic of Korea; 4 KAIST Institute for Health Science and Technology, Daejeon, 34141, Republic of Korea; 5 Department of Biological Sciences, Korea Advanced Institute of Science and Technology, Daejeon, 34141, Republic of Korea; Universitat zu Koln, GERMANY

## Abstract

Multiplexed cyclic imaging in expandable tissue gels has been extensively studied to visualize numerous biomolecules at a nanoscale resolution in situ. Previous studies have employed sparse labels, such as DAPI or lectin staining, as registration markers. However, these sparse labels do not adequately capture the full extent of deformation across the entire region of interest. To overcome this challenge, we propose the use of dense labels, specifically fluorophore *N*-hydroxysuccinimide (NHS)-ester staining, as registration markers to achieve highly accurate image registration. We first tested several NHS-functionalized fluorophores as fiducial markers and identified the proper candidates for three-dimensional (3D) multiplexed cyclic imaging. We analyzed the registration accuracy between DAPI and NHS-ester staining and illustrated that dense label-based registration provides a more accurate registration performance. In the multiplexed imaging of expanded specimens, we observed that repetitive expansion/shrinking processes and chemical treatments for signal elimination can induce 3D nonlinear distortion. This sample distortion can be mitigated by re-embedding the tissue gel or replacing the chemical de-staining process with photobleaching-based signal removal or computational signal unmixing. With such an optimized experimental setup, we demonstrated 3D multiplexed cyclic imaging with nanoscale precision image registration. Finally, we prove that dense biological structures, such as actin, can be used as registration markers to achieve high registration accuracy. We anticipate that the proposed dense labeling strategy will overcome the technical limitations of multiplexed cyclic imaging in expandable tissue gels, offering high-precision registration. We expect it to be widely adopted by the biological and medical communities.

## Abstract 2

Expansion microscopy facilitates multiple rounds of antibody staining and imaging, but this requires high-quality registration of images between rounds. This study develops a superior image registration technique using dense NHS-ester labels for multiplexed cyclic imaging in expandable tissue gels.

## Introduction

Spatially resolved proteomics has significant potential for a variety of biomedical applications, since it can provide deeper insights into biological phenomena and improve diagnostic and prognostic information [1,2]. For precise molecular profiling of biological samples, a high-resolution imaging modality is needed, with strong three-dimensional (3D) multiplexing capabilities. Tissue expansion techniques are attractive solutions for meeting such a need for high-resolution imaging because they visualize nanoscale features below the diffraction limit with the physical expansion of tissue gel (Expansion Microscopy [ExM] [3], Magnified Analysis of Proteome [MAP] [4], etc.). This approach does not necessitate specialized chemicals or equipment because it only modifies the scale of the target sample, making it simple to execute in a typical laboratory setting. Moreover, recent advances in tissue expansion techniques have enabled post-expansion antibody staining [5]. Post-expansion antibody staining enables highly multiplexed imaging by repeated staining, imaging, and fluorescence signal removal [4,5], simultaneously detecting multiple target molecules with enhanced spatial resolution.

When performing cyclic staining and imaging with expanded specimens, the most crucial step is registering images acquired in consecutive imaging rounds [6]. While nuclei or blood vessels are commonly employed as fiducial markers in this process [4], these sparse structures can only encode the degree of spatial deformation where they are present. Because the density of these structures varies even within a single organ type [7], such as the brain, the accuracy of measuring spatial deformation can vary between regions within the same specimen. In addition, because the scale of these structures is in microns, it is challenging to measure the spatial deformation of specimens with nanoscale precision [8]. This limitation is particularly problematic when imaging nanoscale subcellular structures, such as synapses. To address this challenge, nanoscale structures in close proximity to the target proteins, such as synaptic markers for imaging multiple synaptic proteins or fiber endpoints from different cell type markers for imaging fibrous structures, have been utilized as fiducial markers [5,8]. Nonetheless, this approach necessitates the use of numerous fiducial markers to visualize diverse protein structures within a single specimen, thus limiting multiplexing capabilities.

Here, we introduce a dense label-based image registration technique for 3D super-resolution multiplexed cyclic imaging. This offers a substantially high level of registration accuracy, thus addressing the inherent drawbacks of conventional image registration methods based on sparse labels. The dense labeling of the target sample was realized by staining the sample with *N*-hydroxysuccinimide (NHS)-functionalized fluorophores, targeting the amine groups abundant in proteins. Several fluorophore NHS-esters were tested to identify suitable candidates for fiducial markers in multiplexed cyclic imaging. The optimal fluorophore NHS-esters should possess the following characteristics: (1) high fluorescent signals in all types of specimens, (2) heterogeneous structures at a scale of tens of nanometers, (3) resistance to photobleaching, and (4) compatibility with cyclic staining without any spectral shift, even after multiple rounds of staining, imaging, and signal removal. Based on these criteria, we screened various types of fluorophores and found the optimal ones. Subsequently, we illustrated that the registration accuracy obtained using these fluorophores was higher than that achieved using conventional fiducial markers, such as DAPI. Finally, we demonstrate the 3D multiplexed cyclic imaging of an expandable tissue gel with a dense label-based registration strategy, combined with sample treatment processes that effectively minimized sample distortion throughout imaging rounds. In addition, we proved that the accurate registration of the cyclic imaging of expandable tissue gel can be achieved not only with fluorophore NHS-ester staining but also with dense biological structures, such as actin. This finding expands the range of options for achieving precise registration in multiplexed cyclic imaging studies.

## Results and discussion

**Fig 1:**
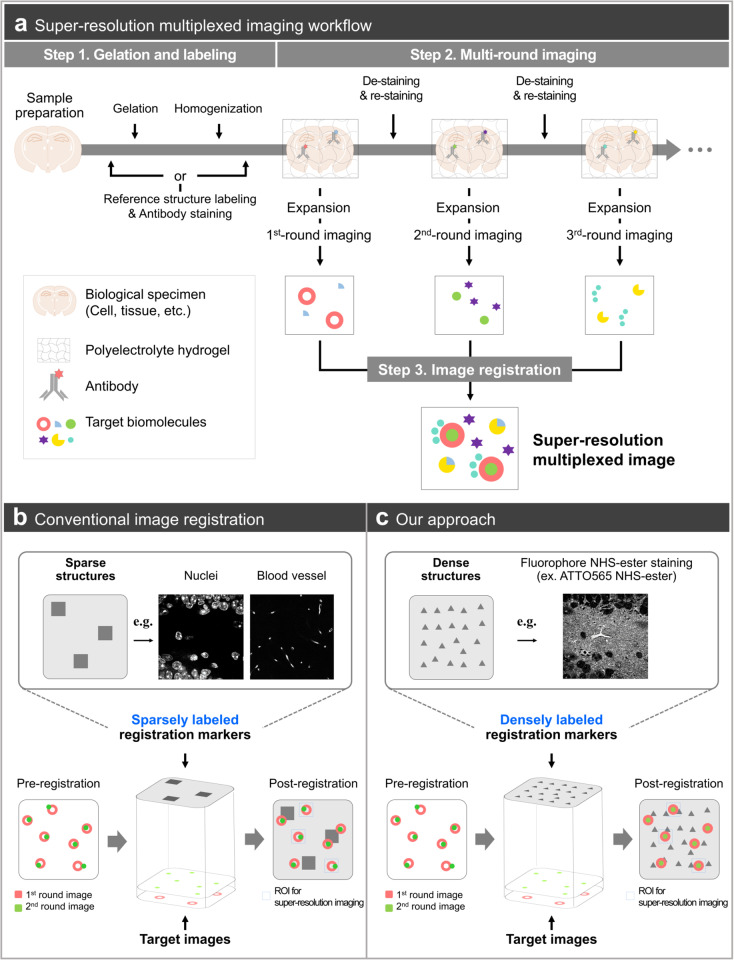
General experimental procedure for super-resolution multiplexed imaging in an expandable hydrogel specimen. **(a)** Stepwise description of multiplexed imaging in an expandable hydrogel specimen. The entire experiment can be divided into three main steps: gelation and labeling, multi-round imaging, and image registration. The first step includes the gelation of the biological specimen, the labeling of biological molecules and fiducial markers, and the physical homogenization of the hydrogel–specimen complex. The sequence of such works can differ depending on the type of expansion method used. The second step is multi-round imaging by repeating the cycle of staining and de-staining. The de-staining step includes all signal elimination approaches, such as antibody stripping, DNA elution, or fluorophore bleaching. After acquiring images from each round, the third step, image registration, is performed to match the distorted pixel coordinates finely. **(b)** Conventional image registration using sparsely labeled markers. Representative sparsely labeled markers are nuclei or blood vessels. An image registered with sparsely labeled markers still includes severe pixel mismatches, especially in the region where fiducial markers are barely displayed. **(c)** Image registration using dense-labeled markers. Fluorophore NHS-ester staining can be utilized as a densely labeled fiducial marker. Since fiducial markers are densely localized in the entire field-of-view, this approach provides relatively high registration accuracy.

Various tissue expansion techniques have been developed for super-resolution imaging. In this study, we adopted the epitope-preserving magnified analysis of the proteome (eMAP), which allows repeated antibody staining after specimen expansion [4,5]. Multiplexed cyclic imaging in eMAP involves the following steps: (1) gelation and specimen homogenization, (2) multi-round staining, imaging, and antibody stripping, and (3) registration of the acquired images (Fig 1a). To conduct the image registration of the multiplexed images acquired from consecutive rounds, image channels containing fiducial markers were essential. Cellular-level structures, such as nuclei, blood vessels, and neurons, have been commonly used as fiducial markers [9]. However, these structures are not universally found in biological specimens at different scales. At the scale of conventional diffraction-limited microscopy, nuclei and blood vessels play the role of landmarks for the successful alignment of nearby structures [10]. However, at the scale of super-resolution microscopy, these cellular-level structures are too sparse to guide nearby sub-cellular structures. In particular, in regions of interest for imaging synaptic structures, cellular-level structures are rarely found, and image registration might be unsuccessful (Fig 1b). To overcome this limitation, in this work, we employed a pan-protein staining approach utilizing NHS-functionalized fluorophores that labeled all amines present in the tissue [11–13]. Because amine groups are distributed universally and densely in tissue at the sub-cellular level, we use these targets as fiducial markers for registering images acquired with super-resolution microscopy using tissue expansion (Fig 1c).

As mentioned above, to evaluate the fiducial markers for 3D multiplexed cyclic imaging, we tested fluorophores based on four criteria: (1) High fluorescent intensity, (2) Uniform and high labeling density, (3) Resistance to photobleaching, and (4) High spectral stability. The staining patterns of each type of fluorophore NHS-ester varied depending on the hydrophobicity of the fluorophores [14,15]. Since the cyclic staining strategy inevitably required multiple rounds, the fluorescent signal of the NHS-ester should be stably visualized across repetitive imaging without significant photobleaching and the spectral shift of emission spectra such as red- or blue-shifts. We initially screened various NHS-esters of 405-nm excitable fluorophores. Although the NHS-esters of 405-nm excitable fluorophores, such as CF 405S, CF 405M, and ATTO 390, showed high labeling density, all of the tested fluorophores exhibited significant photobleaching over multiple imaging rounds. In addition, when excited multiple times by a 405-nm laser during the repeated staining and imaging process, the NHS-ester staining of these 405-nm excitable fluorophores was displayed not only in the 405-nm detection channel (422–468 nm) but also in the 488-nm detection channel (502–540 nm), which infers a spectral red-shift in the emission spectrum (S1 Fig). Such a red shift in the emission spectrum of these fluorophores could impede the use of 488-nm excitable fluorophores for staining proteins in specimens. To identify better candidates for fiducial markers, we screened additional 13 fluorophore NHS-esters (488-nm excitable: Alexa Fluor 488, CF 488A, and CF 514, 561-nm excitable: ATTO 565, ATTO Rho 101, ATTO 594, Cy3, and CF 568, 647-nm excitable: ATTO 633, ATTO 647N, ATTO 680, CF 660R, and CF 680R) that are excitable with other excitation laser wavelengths, such as 488-, 561- and 647-nm (S2 Fig). To investigate the resistance to photobleaching and spectral stability, we acquired images from the same field-of-view (FOV) twice, before and after 5-min illumination with corresponding excitation wavelengths (S3 Fig, see S1 Table for the specific intensity drop rate). The 488-nm excitable fluorophores, such as Alexa Fluor 488, CF 488A, and CF 514, showed a significant drop in fluorescent intensity after the 5-min illumination with a 488-nm laser. ATTO 594, a 561-nm excitable fluorophore, displayed much higher resistance to photobleaching, but initially had bleed-through across 561- and 647-nm detection channels. After the 5-min illumination with the 561-nm excitation laser, its signal in the 647-nm detection channel (660–737 nm) decreased, while its signal in the 561-nm detection channel (572–615 nm) showed a significant increase, implying a spectral blue-shift of the emission spectrum. ATTO 647N also showed higher resistance to photobleaching and initially exhibited a fluorescence signal only within the 647-nm detection channel. However, after 5-minute illumination with the 647-nm excitation laser, its signal was also gradually shown in the 561-nm detection channel, which is used for acquiring images of 561-nm excitable fluorophores, indicating a spectral blue-shift of the emission spectrum (S4 Fig). Such aforementioned red- and blue-shifts of the emission spectra of the fluorophores may result from a variety of interactions, such as changes in charge separation within the fluorophore and conformational changes in the fluorophore, including the fragmentation of dyes by photooxidation [16,17]. The characteristics observed in this experiment, in which brain slices stained with fluorophore NHS-esters were exposed to intense excitation lasers for an extended duration, may differ from those of fluorescent molecules under standard immunostaining and imaging conditions. After thorough consideration of the rest of the 7 fluorophore NHS-ester candidates, we narrowed down the selection to 3 fluorophore NHS-esters: Cy3, ATTO 565, and ATTO 680. To assess the impact of fluorophore NHS-ester staining on antibody staining, brain slices derived from the same mouse were prepared. One-half of a brain slice was stained with one of the selected fluorophore NHS-esters (ATTO 565 NHS-ester), while the other half-brain slice was left without NHS-ester staining. Subsequently, both slices were then stained with identical antibody to observe whether any significant difference was derived from NHS-ester staining. A total of 15 different antibodies were tested, and in all cases, no significant difference in antibody staining quality was observed between the samples with and without fluorophore NHS-ester staining. (see S5 Fig for the antibody compatibility test on the ATTO 565 NHS-ester stained mouse brain slices). This constitutes a promising option for the dense labeling strategy.

Next, we attempted to validate NHS-ester staining-based registration and demonstrate multiplexed cyclic imaging using NHS-ester staining as fiducial markers. However, when performing the multi-cycle 3D imaging of expanded specimens, we encountered an issue with non-linear 3D distortion, as reported previously [18]. Specifically, when expanded specimens underwent repeated cycles of staining and antibody stripping in 1× PBS, followed by expansion in DI water, their images exhibited non-linear 3D distortions. The origin of these distortions remains unclear; however, they might be attributed to deformation of the hydrogels’ bottom surfaces on the glass substrate, with distortion propagating throughout the entire hydrogel. Such non-linear distortion necessitates computationally heavy 3D registration that sometimes required human intervention [18]. To address this issue, we performed subsequent multiplexed imaging in 1 × PBS to minimize z-plane distortion and to achieve highly accurate image registration performance. In this process, the specimens expanded only 2-fold and maintained a consistent size throughput, eliminating the need for computationally intensive 3D registration. To perform the entire process in 1 × PBS, we replaced the antibody stripping process used in the eMAP protocol, which involves repetitive high-temperature treatment in a denaturation buffer containing sodium dodecyl sulfate (SDS) and sodium sulfate, with two approaches. First, we photobleached the antibody signals after each round of imaging instead of stripping the antibodies, as reported previously [19]. Second, we replaced the antibody stripping process with computational signal unmixing, as reported previously [20]. These two alternative approaches significantly improved spatial distortion issues (S6 Fig).

**Fig 2:**
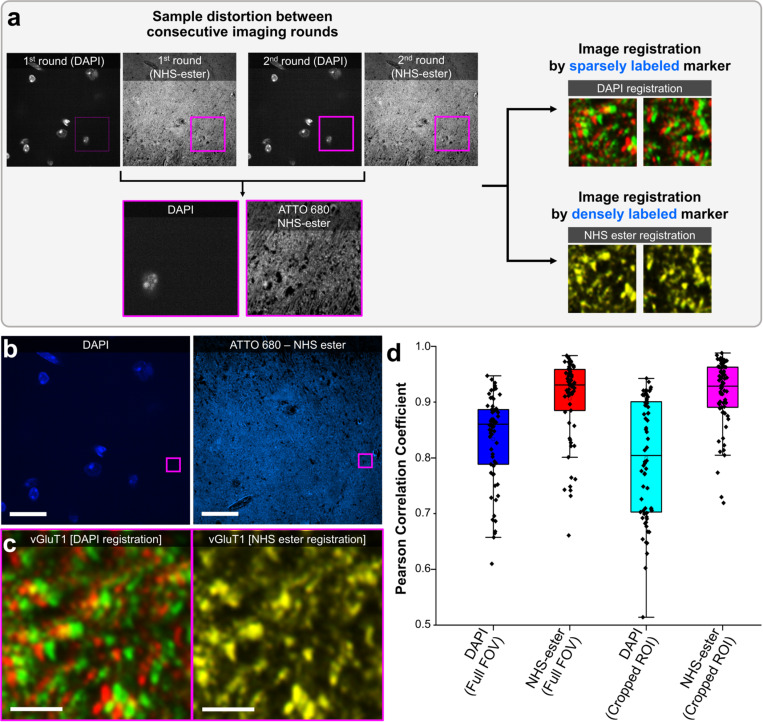
Validation of a dense label-based registration. **(a)** Experimental procedure for validation. The image registration accuracy was estimated from sparsely labeled (DAPI) and densely labeled (ATTO 680 NHS-ester) markers. The first round was stained and imaged with a rabbit anti-vGluT1 antibody and an Alexa Fluor 488 (AF 488)-conjugated goat anti-rabbit secondary antibody. Signal of the first round was photobleached and the second round was stained and imaged with anti AF 488 antibody. Images acquired from the first and second round were finally registered through the DAPI staining channel and the NHS-ester staining channel, respectively. **(b)** Initial region of interest (ROI) with DAPI (blue) and NHS-ester staining (light blue) channels. Target ROI where DAPI signal is barely visible (magenta-highlighted region). **(c)** Magnified view of post-registered vGluT1 fluorescent signal registered by DAPI and NHS-ester, respectively (first round: red, 2nd round green). **(d)** Box plot of Pearson correlation coefficient between DAPI registered images (1st–2nd round) and NHS-ester registered images (1st–2nd round) within initial ROI and magnified target ROI. The brain region where validation has been conducted was between CA3 and dentate gyrus regions. Please see S1 Data for individual numerical values of the Pearson correlation coefficient. Scale bars: (**b**) 20 µm; (**c**) 1 µm. All length scales are presented in pre-expansion dimensions. Number of sample *N* = 6, Number of datapoints *M* = 11 for each sample.

With minimized sample distortion conditions, we compared the registration accuracy of dense labels (fluorophore NHS-esters) with that of sparse labels (DAPI) when used as fiducial markers. The initial assumption was as follows: Since the staining pattern becomes much sparser after expanding the tissue gel, conventional sparse labels, such as DAPI, would provide limited information on the deformation of specimens (S7 Fig). This registration issue might worsen in regions with few cells. In contrast, dense labels, such as fluorophore NHS-ester staining, consistently provided substantial spatial information regardless of the target region of interest, enabling highly accurate image registration performance. We first compared the registration accuracy of these two labels in a small FOV over two consecutive imaging rounds. Mouse brain slices were processed with the eMAP protocol, stained with DAPI and ATTO 680 NHS-ester, and expanded 2-fold in 1 × PBS. Then, they were stained with a primary antibody against pre-synaptic marker (vGluT1) and Alexa Fluor 488-conjugated secondary antibody targeting the primary antibody. After imaging, the fluorescence signals of the specimens were photobleached with prolonged exposure to the excitation laser. The specimens were then stained with a primary antibody against Alexa Fluor 488 and an Alexa Fluor 488-conjugated secondary antibody targeting this primary antibody. To minimize the effect of chromatic aberrations, we stained the sample with an identical fluorophore (Alexa Fluor 488) and acquired images of the 488-nm channel. The two images, one acquired after anti-vGluT1 staining and the other acquired after anti-Alexa Fluor 488 staining, were registered using either DAPI or ATTO 680 (Fig 2a and 2b). For each imaging round, 10-µm thick z-stack images with 1-µm stepsize were acquired from six different samples. Notably, in the regions where the DAPI signal was absent, noticeable pixel mismatches were observed in the post-registered vGluT1 images when the two images were registered using DAPI as a fiducial marker. Conversely, when NHS-ester staining was used as a fiducial marker, it allowed precise registration between the two consecutive round images (Fig 2c; see S8 Fig for more comparison results between DAPI-based and NHS-ester-based registration). Pearson correlation coefficients (PCCs) between the first and second round images were estimated to quantitatively compare the registration accuracy of DAPI staining and NHS-ester staining (Fig 2d; see S9 Fig and S1 Data for a detailed dataplot of PCC among DAPI-based, NHS-ester-based and vGluT1-based registration). The results clearly showed that using dense labels through fluorophore NHS-ester staining as fiducial markers markedly enhanced registration accuracy compared to DAPI staining-based sparse labeling. Furthermore, no significant difference was observed when the NHS-ester-based registration results were compared to those obtained by registering with the target protein image, vGluT1, which was expected to yield the most accurate registration results (see S9 Fig for dataplot of the PCC).

**Fig 3:**
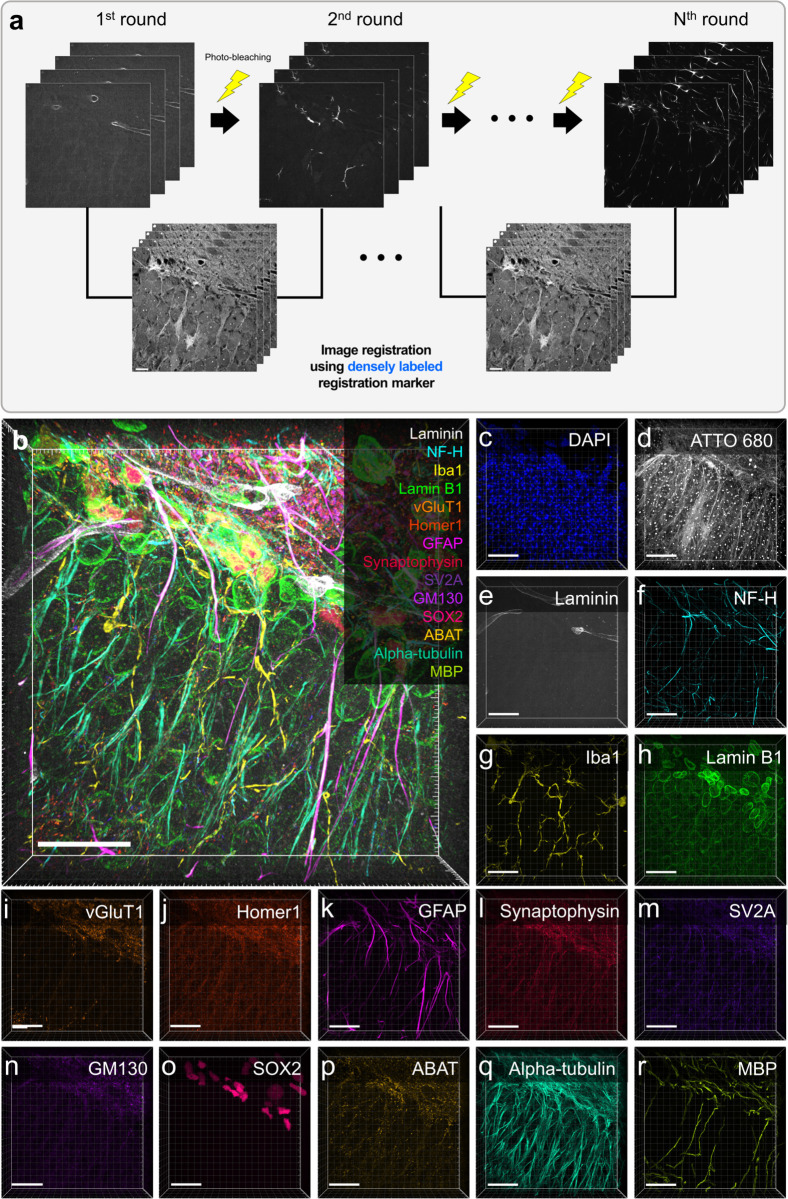
Registration of multiplexed images *via* ATTO 680 NHS-ester staining and photobleaching in an eMAP-processed mouse brain slice. (**a**) Experimental schematic of the registration of multiplexed images. Target proteins for each imaging round were stained, imaged and removed with photobleaching treatment. A pair of images from adjacent rounds are registered by the ATTO 680 NHS-ester staining image. (**b**–**r**) Seven-round cyclic staining images of an eMAP-processed mouse brain slice. (**b**) Merged 3D 16-plex image with 30 µm z-stacks. Blue, DAPI; gray, ATTO 680; white, Laminin; cyan, NF-H; yellow, Iba1; green, Lamin B1; orange, vGluT1; orange-red, Homer1; magenta, GFAP; crimson, Synaptophysin; dark purple, SV2A; purple, GM130; pink, SOX2; gold, ABAT; light green, Alpha-tubulin; green-yellow, MBP. (**c**–**r**) Single-channel images of the target proteins. Scale bars: (**b**–**r**) 20 µm. All length scales are presented in pre-expansion dimensions. Number of sample *N* = 2 acquired from two independent mouse brain slices.

Following the thorough validation of the highly accurate dense label-based registration, we conducted 3D multiplexed cyclic imaging on mouse brain slices through the first approach, which used photobleaching to remove signals. To avoid host species cross-reactivity and to use multiple same host antibodies, we adopted the preformed antibody complex (preassembly) strategy, utilized in our previous study [21,22]. For photobleaching-based multiplexing, we first validated the robustness of the photobleaching strategy and confirmed the absence of antibody crosstalk in the preformed antibody complexes (S10a Fig). Various antibodies were properly stained across multiple rounds of photobleaching, and the preformed antibody complexes visualized the corresponding target protein expression without antibody crosstalk. After validation, we demonstrated multiplexed imaging as follows: eMAP-processed specimens were stained with DAPI and ATTO 680 NHS-ester. Then, antibody staining, 3D imaging, and photobleaching were repeated for seven rounds, with two antibodies stained in each round, resulting in 16-color 3D multiplexed imaging (Fig 3; see S10b and S10c Fig for the prior validation result of photobleaching in an intact mouse brain slice). Notably, glial fibrillary acidic protein (GFAP), the astrocyte marker, showed high spatial colocalization with SRY-box 2 (SOX2) while ionized calcium-binding adapter molecule 1 (Iba1) did not colocalize with either GFAP or SOX2 (S11 Fig). The resulting multiplexed image matched the previously reported protein colocalization patterns [23–27].

**Fig 4:**
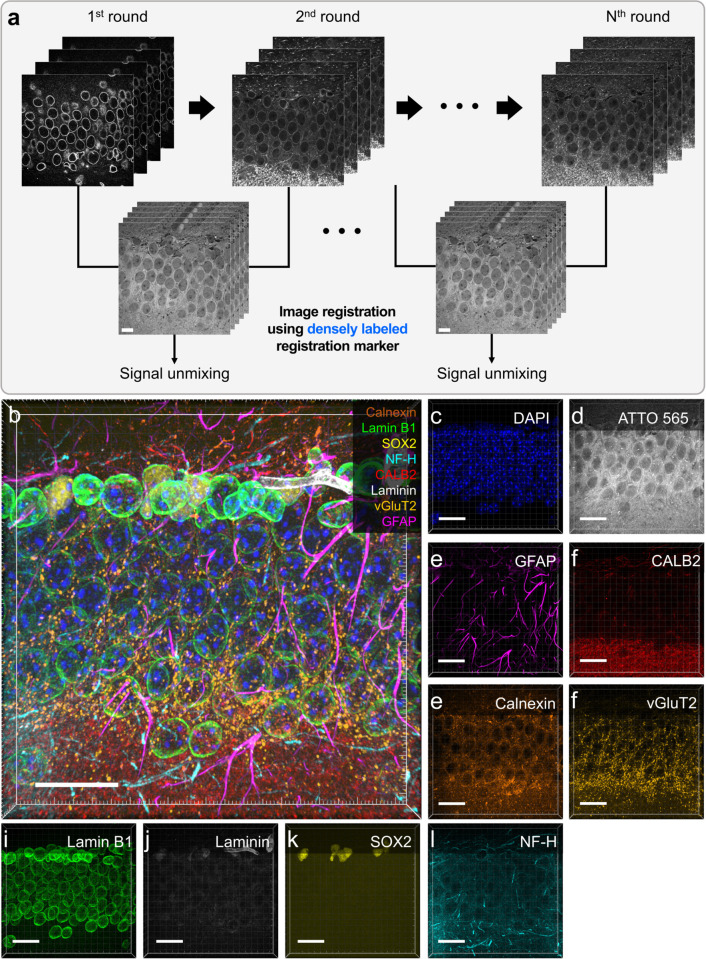
Registration of multiplexed images *via* ATTO 565 NHS-ester staining and signal unmixing in an eMAP-processed mouse brain slice. (**a**) Experimental schematic of the registration of multiplexed images. Target proteins for each imaging round were consecutively stained without signal removal. A pair of images from adjacent rounds are registered by the ATTO 565 NHS-ester staining image. The registered images were unmixed to extract target protein expression. (**b**–**m**) Five-round cyclic staining images of an eMAP-processed mouse brain slice. (**b**) Merged 3D 10-plex image with 20 µm z-stacks. Blue, DAPI; gray, ATTO 565; brown, calnexin; green, lamin B1; yellow, SOX2; cyan, NF-H; red, CALB2; white, laminin; gold, vGluT2; magenta, GFAP. (**c**–**m**) Single-channel images of the target proteins. Scale bars: (**b**–**m**) 20 µm. All length scales are presented in pre-expansion dimensions. Number of sample *N* = 6 acquired from two different mouse brain slices.

We next demonstrated 3D multiplexed cyclic imaging using the second approach, which replaced the antibody stripping process with computational signal unmixing employing the PICASSO technique [21]. In this approach, we consecutively stained the next round of target antibodies without any additional signal removal steps [20]. Then, the *N* + 1th round image was unmixed with the *N*th image using the blind unmixing algorithm reported in the previous works [20,21]. To quantitatively assess the accuracy of signal unmixing, we calculated the PCC between the unmixed output images and the corresponding ground-truth images (S12 Fig; see S2 Data and the Experimental Section for details of the experimental design). Brain slices were consecutively stained for two or three rounds without expansion, followed by image acquisition and signal unmixing. The PCC between the unmixed images and their respective ground-truth images was then computed, yielding a value of approximately 0.98, indicating a high degree of correspondence with the ground-truth images. Using this approach, we demonstrated 10-color 3D multiplexed imaging (Fig 4).

To compare the registration accuracy between dense label-based and sparse label-based registration within an approximately 4× fully expanded state while minimizing the variation in the expansion factors throughout the rounds, we re-embedded an expanded tissue gel in a neutral gel and performed cyclic imaging. In this experiment, we used a proteinase-based ExM technique demonstrated in 2015 [3]. After re-embedding, we conducted a series of steps: hybridizing fluorophore-conjugated oligonucleotides (imager DNA) to the gel-anchored oligonucleotides (tertiary DNAs), imaging, and de-hybridizing the imager DNA from the hydrogel. This cycle was repeated three times. To precisely measure the registration error not affected by chromatic aberration, we used the same fluorophore to label the same DNA in different imaging rounds. Images were then registered using either the DAPI or fluorophore NHS-ester channel as fiducial markers, and registration errors were measured by analyzing line profiles. We found that registration using DAPI as a fiducial marker yielded a registration error larger than 50 nm, especially when the nucleus density was low, even though the gel was re-embedded in a neutral gel and did not undergo repeated shrinking and expansion (S13a and S13b Fig). This expanded brain slice underwent non-linear deformation, resulting in a registration error where the nucleus was absent (S13c and S13d Fig). However, when Cy3 NHS-ester staining was used as a fiducial marker for the repetitive imaging of an expanded mouse brain slice, a registration error of less than 10 nm was achieved, as shown in S13e–S13h Fig (see S3 Data for detailed analysis of the line profiles). For the further quantitative evaluation of registration accuracy, we cropped several sub-regions of interest (ROIs) of Bassoon and GFAP structures acquired from the first and second rounds and the second and third rounds, respectively (S13i–S13l Fig). PCCs were calculated to measure two variables’ linear correlation. The PCCs acquired from the Bassoon and GFAP images both showed that NHS-ester-based registration outperformed DAPI-based registration by approximately 20% (S13m Fig, see S4 Data for detailed analysis of the accuracy of computational signal unmixing).

After validating the high registration accuracy of dense label-based registration, we turned our attention to exploring alternative dense labels as registration markers. While fluorophore NHS-esters visualize diverse structures, their lack of molecular specificity prompted us to consider dense biological structures for this purpose. We specifically chose actin as our dense label for registration. Actin fulfills the four essential requirements for effective registration markers. First, it exhibits high heterogeneity on a scale of tens of nanometers, which helps achieve precise registration [28]. Second, actin is present in all types of specimens, making it a versatile choice for various biological samples [29]. Third, actin demonstrated uniform expression for all the specimens, ensuring consistent and reliable labeling for registration [29]. Last, actin is compatible with cyclic staining in expanded specimens, allowing repeated imaging cycles without compromising the registration process [30]. To validate this idea and showcase its potential applications, we performed multiplexed imaging in cultured cells using simple DNA-based barcoding. To achieve this, we employed the actin-ExM technique, which we recently developed for simple actin staining and expansion [30].

**Fig 5:**
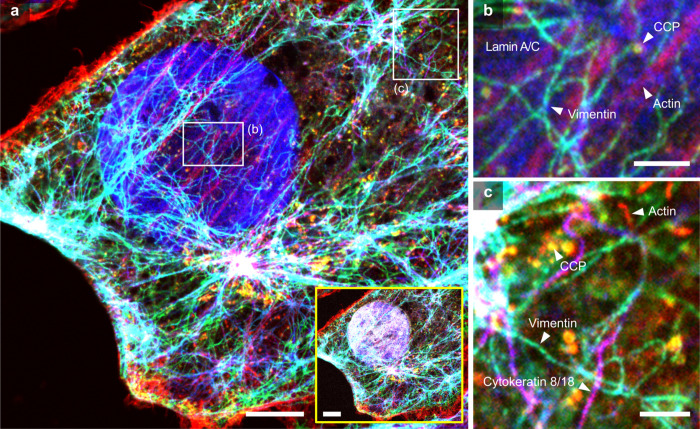
Registration of multiplexed images *via* actin staining in expanded cultured cells. (**a**) Composite image generated by registering two images acquired from the first and second imaging rounds. Phalloidin-labeled actin fibers were used as fiducial markers. The yellow box displays six-color images, including the DAPI channel. Gray, nucleus; red, actin; cyan, vimentin; blue, laminA/C; yellow, CCP; and magenta, cytokeratin 8/18. (**b**–**c**) Magnified views of the boxed region in a. Scale bars: (**a**) 2 µm; (**b**–**c**) 500 nm. All length scales are presented in pre-expansion dimensions. Number of sample *N* = 1 acquired from a single substrate.

Briefly, cultured cells were labeled with fluorescein isothiocyanate (FITC)-conjugated phalloidin, primary antibodies against FITC, oligonucleotide-conjugated secondary antibodies against primary antibodies, and four proteins: vimentin, lamin A/C, clathrin heavy chain (CCP), and cytokeratin 8/18. Following tertiary DNA hybridization, the cells were expanded 4.2-fold using previously established pro-ExM protocols [3,31]. The expanded specimen was then embedded in an uncharged polyacrylamide gel to maintain its expanded state during the two-round imaging process. We then repeated the hybridization and dehybridization of the fluorophore-conjugated oligonucleotides to generate a distinct color barcode for each protein. To analyze the barcoded image completely, we registered two images taken from sequential rounds using the actin channel as a fiducial marker and found that all proteins were clearly designated by their own color codes (Figs 5a, S14a, and S14b). For example, vimentin was labeled with Alexa Fluor 488 in the first round (displayed in blue) and the second round (displayed in green), showing cyan-labeled fibers in the resultant image. CCP was also labeled with Alexa Fluor 488 in the second round (displayed in green) but with ATTO 565 in the first round (displayed in red), resulting in yellow-labeled ring structures in the cell (Figs 5a, S14c, and S14d). As visualized in Fig 5b and 5c, we observed single fibers of different types of cytoskeleton—vimentin and cytokeratin—in assorted colors after a 4.2-fold expansion. While the actin channel was used as a fiducial marker, it also visualized relative spatial distribution information with other structures as an important cytoskeleton.

## Conclusions

In this work, we propose a dense label-based image registration strategy for multiplexed cyclic imaging in expandable tissue gel by staining with either fluorophore NHS-ester or a densely distributed biological structure. Conventional sparse labels, such as DAPI or lectin staining, provide spatial information for limited regions where only their signals exist. Thus, image registration accuracy in blank regions or at subcellular scales is not guaranteed. Our strategy aimed to utilize universally and densely expressed labeling to overcome the drawbacks of conventional sparse labeling methods, ensuring highly accurate image registration across the entire region of interest. Given the unique properties of each fluorophore NHS-ester, we first screened a couple of fluorophore NHS-esters to find the most appropriate candidate. We thoroughly considered several factors to identify the characteristics of each fluorophore NHS-ester, such as labeling density, fluorescent intensity, hydrophobicity, resistance to photobleaching, and photostability. For our dense labeling strategy, the ideal characteristics entail a high-density expression pattern with a sufficient level of persistence of fluorescent intensity across multiple imaging rounds, without any significant photobleaching or spectral shifts. Following our screening process, we identified three fluorophore NHS-esters, that are, Cy3, ATTO 565, and ATTO 680, as promising options for 3D multiplexed cyclic imaging.

Super-resolution imaging using combination of tissue expansion and confocal microscopy still has technical limitation in z-plane resolution. The non-linear 3D distortion derived from the repetitive expansion and shrinkage of the sample, results in the deterioration of the image registration performance. We further stabilized tissue samples by re-embedding the tissue gel for additional mechanical support or employing photobleaching-based signal removal or computational signal unmixing to reduce the cost of 3D registration. The registration accuracy between sparse labeling (DAPI staining) and dense labeling (NHS-ester staining) was compared using the cyclic staining of identical synaptic proteins in the first and second rounds. The sequentially acquired images were registered through a corresponding fiducial marker channel. Post-registration analysis demonstrated that dense label-based registration achieved significantly higher image registration accuracy, especially in regions where DAPI signals were rarely displayed. With thorough validation and optimized experimental setup, we successfully demonstrated 3D multiplexed cyclic imaging through either photobleaching-based or signal unmixing-based approaches in a 2-fold expanded mouse brain slice with high-precision image registration. For robust cyclic imaging, the samples were maintained in a 2-fold expansion state to minimize possible spatial distortion and orientation mismatch. However, this approach has the drawback of a potentially lower spatial resolution compared to the 4-fold fully expanded state. We further quantitatively estimated the registration accuracy between DAPI and NHS-ester staining in a 4-fold expanded mouse brain slice by line profiling the same structure from consecutive imaging rounds and measuring the PCCs. The post-registration analysis revealed a registration error of over 50-nm for DAPI staining, while NHS-ester staining achieved fine registration with less than a 10-nm registration error.

We also demonstrated the application of a dense label-based registration strategy with densely distributed biological structures instead of fluorophore NHS-ester staining. This result indicates that such a dense label-based registration strategy is not exclusively restricted to the chemical staining of fluorophore NHS-ester but can be applied more broadly to densely dispersed structures. A common limitation of all cyclic staining methods is the need to sacrifice one laser channel as a registration marker. This results in the loss of one fluorescence channel, thereby reducing imaging efficiency. However, if densely distributed structures can be reliably secured, it might be possible to integrate phase contrast imaging or other methods that do not require sacrificing a fluorescence channel. Such an approach would increase the number of channels for simultaneous use, ultimately enhancing the multiplexing capability of the imaging system. Integrating this dense labeling strategy with signal amplification techniques using nanoparticle growth (S15 Fig), the repetitive staining of target molecules [32], or the tyramide signal amplification technique [33] would improve the visualization of sparsely expressed proteins. Furthermore, the compatibility of diverse fluorophores with proteinase K-based ExM and eMAP offers an opportunity to enhance multiplexing capabilities by integrating them with advanced multiplexed imaging techniques [21,34]. Due to this exhaustive demonstration, we believe the proposed dense labeling strategy can provide a substantially higher level of precise image registration than that of conventional sparse labeling and will find applications in biomedical fields associated with multiplexed cyclic imaging.

## Experimental section

### Ethical statement

All of the following procedures involving animals were approved by the Korea Advanced Institute of Science and Technology Institutional Animal Care and Use Committee (KAIST-IACUC; KA2019-64, KA2025-063). All young adult male and female C57BL/6 mice were housed in a 12-h light/dark cycle with unrestricted access to food and water in a specific pathogen-free facility of the KAIST Laboratory Animal Resource Center.

### Materials

All the chemicals and non-chemical supplies used in this study are listed in S2 Table. The concentrations of the labeling agents, including the fluorophore NHS-esters, antibodies, phalloidin, DAPI, and DNA oligos, are listed in S3 Table. DNA sequences and the list of imager DNAs for sequential imaging are listed in S4 and S5 Tables.

### Cell culture

BS-C-1 cells were cultured in Culturewell chambered coverglasses in a minimum essential media supplemented with 10% fetal bovine serum, 1% penicillin-streptomycin, and 1% sodium pyruvate. Cells were cultured at 37 °C in humidified 5% CO_2_.

### Mouse brain slice preparation

Mice aged 8–14 weeks were anesthetized with isoflurane and transcardially perfused with ice-cold 4% paraformaldehyde (PFA) in 1× phosphate-buffered saline (PBS). Brains were extracted and incubated in the same solution at 4 °C for 4 h, and sliced into 150-μm-thick slices on a vibratome (Leica VT1000S). The slices were stored in 0.1 M glycine and 0.01% [w/v] sodium azide in 1× PBS at 4 °C before use.

### Selection of fluorophore NHS-ester as a registration marker for cyclic imaging

For the selection of fluorophore NHS-esters, it was confirmed that, except for the 405-nm excitable fluorophore NHS-esters, all other fluorophores provide sufficiently strong fluorescence signals and labeling densities. However, the primary property required for a fiducial marker is the ability to maintain stable fluorescence signals across multiple imaging rounds that are suitable for cyclic staining applications. While fluorophore NHS-esters with severe photobleaching still exhibited a sufficient level of fluorescence signal, the decrease in fluorescence intensity was relatively rapid. In contrast, the fluorophore NHS-esters we selected showed only minimal photobleaching, making them more suitable for cyclic staining.

### Secondary Fab fragment antibody-fluorophore conjugation and preformed antibody complex (preassembly) staining

For antibody screening and multiplexed cyclic imaging in eMAP-processed mouse brain slices, fluorophore-conjugated Fab fragment secondary antibodies were used for better screening performance without problems with host species reactivity [22]. More than three-quarters of the entire screened antibody panel was from the same host, and each three of them was simultaneously stained and imaged by simply assigning three distinguishable fluorophore-conjugated Fab fragment secondary antibodies such as CF 488A, CF 568, and CF 633. The conjugation procedure was described as follows: The base solution was composed of 90 μL of unconjugated Fab fragment antibody solution and 10 μL of 1 M sodium bicarbonate (pH 8.3). The base solution was mixed with either a 9-fold molar excess of Alexa or CF NHS-ester or 3-fold molar excess of ATTO NHS-ester stock in dimethyl sulfoxide (DMSO), respectively. Antibody-fluorophore conjugation was performed during an hour-long incubation at room temperature (RT; 20–25 °C) in darkness. Conjugated antibodies were purified by NAP-5 gel filtration, and additional concentration work was conducted with centrifugal filters. For preformed antibody complex (preassembly) staining, each rabbit-derived primary antibody was first incubated with a fluorophore-conjugated goat anti-rabbit secondary Fab fragment. Subsequently, any unreacted secondary Fab fragments were blocked using a normal rabbit serum buffer. This ensured that only the preformed primary antibody–secondary Fab fragment complex bound specifically to their target antigen, preventing any non-specific interactions with other rabbit-derived primary antibodies.

### Secondary antibody-DNA oligo conjugation for sequential imaging

The conjugation of DNA to secondary antibodies was conducted using Solulink reagents, following the step-by-step instruction available at http://expansionmicroscopy.org/. Specifically, 5′-end amine-modified DNA oligonucleotides were reacted with a sulfo-S4FB crosslinker to form a 4FB-oligonucleotide complex. Then, secondary antibodies were reacted with a S-HyNic crosslinker to form an antibody-HyNic complex. Finally, 4FB-oligonucleotides and antibody-HyNic complexes were then reacted to form DNA-antibody conjugates.

For modifying DNA oligos, purified DNA was suspended in phosphate buffer (150 mM NaCl, 100 mM Na_2_HPO_4_, pH 7.8) to a concentration of 25 μg/μL. The sulfo-S4FB crosslinker was prepared at a concentration of 25 μg/μL in DMSO. The sulfo-S4FB crosslinker and the DNA oligos were then mixed at a molar ratio of 15:1 for at least 2 h. The reactants were centrifugally filtered to completely remove the unreacted sulfo-4FB. After purification, the resultant 4FB-DNA concentrate was diluted in phosphate buffer (150 mM NaCl, 100 mM Na_2_HPO_4_, pH 6.0) to a few mM.

For modifying antibodies, 1 mg of S-HyNic crosslinker was completely dissolved in 350 μL of dimethylformamide. Then, the secondary antibodies and S-HyNic solution were mixed at a volume ratio of 50:1, and the antibody/S-HyNic solution was incubated for at least 2 h at RT. After the reaction, the buffer of the reactant was exchanged for phosphate buffer (150 mM NaCl, 100 mM Na_2_HPO_4_, pH 6.0) using a spin column.

4FB-DNA and antibody-HyNic were then mixed, and 10× Turbolink Catalyst Buffer with a volume of 1/9 of the total volume of the mixture was added. The reaction solution was incubated for at least 2 h at RT. After the reaction, the product was diluted with 1× PBS and then purified with a centrifugal filter to completely remove unreacted DNA from the reactant. The DNA-conjugated antibodies were then diluted with 1× PBS and stored at 4 °C.

### Sequential imaging of expanded mouse brain slices

For the results shown in S13 Fig, brain slices were first blocked and permeabilized with normal donkey serum (NDS) blocking buffer (5% NDS, 0.2% Triton X-100, 1× PBS) for 2 h, and then treated with 0.1 mg/mL AcX in 1× PBS, followed by washing in 1× PBS three times for 30 min each time. Then, brain slices were incubated with primary antibodies in NDS blocking buffer for 3 h, followed by washing in NDS blocking buffer four times for 30 min each time. Next, brain slices were incubated with DNA-conjugated secondary antibodies in hybridization buffer (5% NDS, 0.2% [w/v] Triton X-100, 2× saline sodium citrate buffer [pH 7.0], 0.2 mg/mL sheared salmon sperm DNA) overnight, followed by washing in hybridization buffer four times for 30 min each time. Brain slices were then incubated with 1 ng/μL DNAs with 5′ acrydite modification (which we designated “tertiary DNAs”) in hybridization buffer overnight, then washed in hybridization buffer four times for 30 min each time.

For gelation, tertiary DNA-stained brain slices were incubated in pre-gel solution (8.625% [w/w] sodium acrylate, 2.5% [w/w] acrylamide, 0.2% [w/w] *N*,*N*-(1,2-dihydroxyethylene) bisacrylamide [DHEBA], 1.865 M NaCl, 1× PBS) overnight at 4 °C with gentle shaking. After incubation, brain slices were incubated in gelation solution (8.625% [w/w] sodium acrylate, 2.5% [w/w] acrylamide, 0.2% [w/w] DHEBA, 1.865M NaCl, 1× PBS, 0.2% [w/w] APS, 0.2% [v/v] TEMED, 0.01% [w/w] H-TEMPO) twice at 4 °C for 30 min each time. Then, the slices were subjected to gelation and digestion as previously described in the iExM protocol [35]. Digested gels were then briefly rinsed with 0.1% PBST (0.1% Trtion X-100 in 1× PBS) three times and stained with Cy3 NHS-ester in 0.1% PBST at a concentration of 1 μg/mL overnight at 4 °C with gentle shaking. After staining, gels were washed with 0.1% PBST three times for 30 min each time. The gels were then washed with an excess volume of deionized water for expansion until the size of the gel plateaued. Expanded gels were then re-embedded as previously described in the iExM protocol.

For sequential imaging, re-embedded gels were pre-hybridized in DNA hybridization buffer (4× SSC, 20% [v/v] formamide) for 30 min. Pre-hybridized specimens were incubated with imager DNAs at a concentration of 1.5–2 ng/μL in hybridization buffer, and washed in hybridization buffer six times for 1 h each time, followed by washing once in 1× PBS for 30 min. Washed gels were then incubated with DAPI diluted in 1× PBS for 1 h, washed with 1× PBS twice for 30 min each time, and then imaged. The imaged gels were treated with an excess volume of 50% (v/v) formamide twice at 60 °C for 1 h each time to fully dehybridize the imager DNAs. Gels were then pre-hybridized in hybridization buffer again for the next round of imager DNA hybridization. The aforementioned hybridization and imaging procedures were repeated to acquire multiple rounds of sequential images.

### Cyclic imaging of eMAP-processed mouse brain slices

We followed the eMAP protocol [5] but included an additional fluorophore NHS-ester staining step. Brain slices were incubated in 0.1% PBST for 1 h at 4 °C for permeabilization, then stained with ATTO 565 or ATTO 680 NHS-ester in 0.1% PBST at a concentration of 1 μg/mL for 2 h at 4 °C, followed by washing with 0.1% PBST for 1 hour three times at 4 °C. Then, samples were incubated in eMAP solution (30% acrylamide, 10% sodium acrylate, 0.1% BIS, and 0.03% VA-044 [w/v] in 1 × PBS) overnight in darkness at 4 °C. The incubated brain slice was mounted on a slide glass and covered by coverslip with a gap made by spacers on both the left and right edges. The mounted sample was incubated with nitrogen gas overnight at 50 °C. After gelation, excess outer gel was trimmed, and the sample was incubated in hydration solution (0.1% [w/v] Triton X-100, 0.02% [w/v] sodium azide in 1× PBS) overnight in darkness at 37 °C. We cleared the brain tissue gel by incubating it in clearing solution (6% [w/v] SDS, 50 mM sodium sulfite, 0.02% [w/v] sodium azide) for 6 h at 37 °C, followed by the denaturation process for 10 min at 95 °C within an identical clearing solution. The final eMAP-processed brain tissue gel was thoroughly washed with 0.1% PBST for 1 h, three times at RT to ensure washing out of SDS residue.

For immunostaining and cyclic imaging, the cleared brain tissue gel was incubated in a blocking buffer for 1 h at RT. We mounted the tissue gel on the 0.01% poly l-lysine coated slideglass to prevent sample drift. Sample boundary was blocked with hydrophobic pen. Then, we immunostained the brain tissue gel with primary antibody-secondary Fab fragment preformation complexes overnight in darkness at RT, followed by washing with 0.1% PBST for 30 min three times at RT. For photobleaching-based signal removal strategy, the sample was illuminated by excitation laser wavelength for 10–30 min to three-dimensionally photo-bleach the fluorescent signal of prior round and then, the sample was immunostained for the following round. Time spent for photobleaching might vary depending on the photostability of each fluorophore. For signal unmixing strategy, after each round of imaging, the sample was immunostained for the following round without erasing prior round signals. Signal unmixing-based cyclic imaging might require highly stable microscope stage to acquire images from same z-stacks across multiple rounds. Such immunostaining/imaging/(photobleaching) cycle was repeated iteratively until the last imaging round.

### Sequential imaging of expanded cultured cells

For the results shown in Fig 5, cells were washed three times with 1× PBS and fixed for 10 min with 4% PFA in 1× PBS. The cells were then incubated for 10 min with 0.1 M glycine in 1× PBS and washed three times with 1× PBS. The fixed cells were incubated for 30 min in a blocking buffer, then stained for 60 min with primary antibodies with fluorescein (FITC)-conjugated phalloidin at a concentration of 1.65 μM diluted in a blocking buffer, and then washed three times with a blocking buffer. Stained cells were processed identically to the secondary antibody staining, tertiary DNA hybridization, AcX treatment, gelation, digestion, re-embedding, and sequential imaging procedures of the brain slices, except for the incubation time (DNA-conjugated secondary antibody staining, 30 min; tertiary DNA hybridization, 30 min; AcX treatment, 1 h; monomer incubation, 15 min; gelation, 1.5 h; digestion, overnight; imager DNA hybridization; 30 min; dehybridization, 30 min). In this study, we utilized such a barcoding strategy with a limited number of protein targets to avoid barcoding error issues by ensuring that target proteins do not spatially overlap. While this approach partially overcome the barcoding error problem, it cannot fundamentally resolve the technical limitations. Thus, there remains a clear limitation in increasing the number of rounds and protein targets through barcoding strategy [36,37]. This study aims to demonstrate that multiplexing methods such as barcoding can achieve a certain level of multiplexing, but their application remains limited due to the challenges discussed above.

### Computational signal unmixing process

We adopted the signal unmixing code to separate mixed signals between two adjacent rounds [20,21]. Cyclic staining was performed without de-staining to acquire images, consecutively stacking target protein expressions. In this procedure, a pair of input images from consecutive rounds, displaying “A” and “A+B”, were used. The unmixing process was performed using a custom PICASSO unmixing code [21], which retrieves an image of protein “B” from the mixed input image “A+B”. Such a signal unmixing approach enabled multiplexed cyclic volumetric imaging without any significant sample distortion derived from either repetitive expansion shrinkage or chemical treatment for signal removal. Slight differences in brightness do not pose major issues. However, if a target is excessively bright to the extent that it obscures the expression of targets in previous or subsequent rounds, it would be recommended to position the excessively bright target in the later rounds.

### Microscopy and imaging

For the imaging of expanded hydrogels, the hydrogels were attached to 48 × 60 mm cover glasses coated with 0.01% poly l-lysine to prevent the drift of the hydrogels during imaging. The cover glasses were treated for 30 min with 0.01% poly l-lysine in drying oven. The hydrogels were then placed on the cover glasses and imaged using confocal microscopy. The resulting images in Figs 2**–**4 and related supplementary figures were acquired with imaging the expandable tissue gel in 1× PBS buffer, maintaining ~2× expansion state while images in Figs 5, S13, and S14 were acquired with imaging the re-embedded tissue gel in DI water for ~4× fully expanded state. In this study, we performed the quantitative analyses and the multiplexed cyclic imaging within two imaging conditions; Nikon C2 Plus point-scanning confocal microscope equipped with four excitation lasers (405, 488, 561, and 647 nm) and Andor DragonFly spinning-disk confocal microscope equipped with four excitation lasers (405, 488, 561, and 647 nm). Each laser channel has a detection range as follows: 405-nm channel (422–468-nm), 488-nm channel (502–540-nm), 561-nm channel (572–615-nm), and 647-nm channel (660–737-nm). All the images in this study were acquired with either 40× water immersion objective lens with 1.15 numerical aperture (NA) or 60× water immersion lens with 1.00 NA. Multiplexed images in the figures were adjusted using the “Auto adjust” function of ImarisViewer software (ImarisViewer 10.0.1) and ImageJ (Fiji) to improve visibility. For the images with excessively high brightness values, the maximum intensity scale value was increased after auto adjustment to match the brightness to a similar level as the other target protein images.

### Determination of the expansion factor

The expansion factors were determined by measuring the gel sizes before and after expansion. Additionally, the same structural landmarks were imaged before and after expansion, and the expansion factor was calculated using these landmarks. The expansion factor calculated using landmarks was consistent with the expansion factor calculated using gels.

### Image registration for each imaging round

The ImageJ plugin “bUnwarpJ” is utilized for all the image registration steps in this study. In detail, the fiducial marker image in the first imaging round (either sparse labeling or dense labeling) is assigned as the reference image, and then such images in the following imaging rounds are processed to obtain the registration vector map. Target imaging channels are then registered by the acquired vector map, successfully solving the pixel mismatch issue among the imaging rounds.

Generally, the registration software is executed without manual setting of separate anchor points, allowing for automatic registration. However, in some cases, even though the registration marker images between rounds visually appear nearly identical, it has been observed that the registration vector map may become slightly distorted. The exact cause of this issue has not been clarified, but it has been confirmed that in such cases, manual anchor points (2–3 points) can be designated, which allows the registration to be successfully completed without further issues. The need for manual anchor point specification occurs in about 1–2 stacks out of every 20 stacks, or in majority of cases where no issues are observed, the automatic registration was sufficient.

### Chromatic aberration correction in multi-channel image registration

The underlying assumption of our approach is that distortion remains the same across all channels, allowing the distortion vector map measured from the registration channel to be applied to other channels without additional processing. However, if distortion vector maps depend on wavelength, it may be necessary to measure the chromatic aberration correction map and apply both the aberration and distortion maps to the antibody channels. To mitigate the impact of chromatic aberration on image registration, a potential approach involves performing identical staining across all laser channels, measuring the degree of chromatic aberration, and acquiring a registration vector map across all laser channels as prior knowledge. This vector map would correct chromatic aberration between channels by registering images obtained from each laser channel. Before conducting registration using a reference channel, a preliminary first-round registration could be applied to the other laser channels using the chromatic aberration correction vector map.

### Quantitative analysis for assessing image registration and signal unmixing

To quantitatively evaluate image registration accuracy, the similarity between the 1st round image and the registered 2nd round image was assessed. Additionally, to quantitatively evaluate signal unmixing accuracy, the similarity between the ground-truth image of the target protein expression and the unmixed image was measured. For both quantitative assessments, the PCC was utilized as the metric to quantify the similarity between the two images. The PCC is a suitable parameter for measuring the similarity between two different images because it quantifies the linear relationship between their pixel values, offering several advantages as follows:

**Quantifies the degree of similarity**: The Pearson’s correlation coefficient (r) ranges from −1 to +1, where +1 indicates a perfect positive linear relationship, −1 indicates a perfect negative linear relationship, and 0 indicates no linear correlation. This allows for a clear, numeric understanding of how closely related the two images are in their pixel intensity distributions.

**Sensitivity to intensity patterns**: Because images often contain varying intensity patterns based on structure, texture, or features, Pearson’s correlation is useful for assessing whether two images share similar intensity variations across corresponding pixels.

**Robustness to global brightness and contrast differences**: The PCC is normalized, meaning it is less affected by global changes in brightness or contrast between the two images. This is crucial in situations where two images (unmixed image and ground-truth image) might differ in image acquisition conditions such as the laser channel, laser power, exposure time, or contrast, but still have the same underlying features or patterns. By focusing on the relative relationships between pixels, rather than their absolute values, the Pearson correlation can still provide a meaningful measure of similarity.

Thus, the Pearson correlation is commonly used in image registration, feature matching, and similarity analysis because it effectively captures the degree of linear relationship between the images, independent of global intensity differences.

### Experimental design for assessing signal unmixing accuracy

Quantitative analysis of signal unmixing accuracy was conducted with two different sample types (10-μm-thick cryo-sectioned brain slices and 150-μm-thick brain slices;  total number of specimens, n = 5) and two different staining approaches (indirect staining and preassembly staining). For the indirect immunostaining approach, the first round involved immunostaining with a chicken anti-myelin basic protein (MBP) primary antibody, followed by a goat anti-chicken secondary antibody conjugated with CF 488A fluorophore (488‑nm channel). In the second round, immunostaining was performed with a rabbit anti-nucleolin primary antibody, followed by a goat anti-rabbit secondary antibody conjugated with both CF 488A (488‑nm channel) and CF 568 (561‑nm channel). The 488‑nm channel image from the second round, which serves as an input mixed image for signal unmixing, contains signals from both MBP and nucleolin. The 561‑nm channel image, displaying only nucleolin, was used as the ground-truth image. For the preassembly staining approach, the first round involved immunostaining with a rabbit anti-calnexin primary antibody pre-assembled with a goat anti-rabbit secondary Fab fragment conjugated to CF 568 (561‑nm channel). In the second round, immunostaining was performed with a rabbit anti-nucleolin primary antibody pre-assembled with a goat anti‑rabbit secondary Fab fragment conjugated to CF 488A (488‑nm channel) and CF 568 (561‑nm channel). The third round employed a rabbit anti-GFAP primary antibody pre-assembled with a goat anti‑rabbit secondary Fab fragment conjugated to CF 568 (561‑nm channel) and CF 640R (647‑nm channel). The 561‑nm channel of the second round contains signals from both calnexin and nucleolin and was used as the input for signal unmixing. Similarly, in the 561‑nm channel of the third round, the mixed image comprising calnexin, nucleolin, and GFAP was used for signal unmixing. Ground‐truth images were provided by the single-channel nucleolin image acquired in the 488‑nm channel and the GFAP image acquired in the 647‑nm channel. Generally, input images for unmixing exhibit a relationship such as “A” and “A+B”. The output of the unmixing process is the image of “B”. The PCC was calculated between the output image and the ground-truth image of “B”. It is important to note that, even under optimal unmixing conditions, discrepancies between the unmixed and ground‐truth images may still occur due to factors such as chromatic aberration, shot noise, and variations in the staining patterns of the same primary antibody when used with different fluorophores. To evaluate how effectively the true target protein features are retrieved through unmixing, both sets of images were resized before calculating the PCC between the output unmixed image and the ground-truth image (see S2 Data for details of the experimental design). From the seven images, bright spots potentially caused by non-specific antibody binding were observed. In these cases, images were cropped to exclude the influence of non-specific binding on the assessment of signal unmixing accuracy. However, the unmixing accuracy calculated from the uncropped images showed no significant difference compared to the cropped versions.

### In situ silver growth

The cells were post-fixed with 1% GA in 1× PBS for 10 min, and then washed with DI water three times for 5 min each. The post-fixed cells were washed with 0.02 M sodium citrate (Sigma) three times for 5 min each and in situ silver development was performed with HQ silver (Nanoprobes) according to the manufacturer’s protocol, followed by rinse with DI water three times for 1 min.

## Supporting information

S1 Fig Fluorescence intensity measurement and spectral shift of 405-nm excitable fluorophore NHS-esters. **(a)** Images acquired from the CF 405S NHS-ester stained sample, showing low fluorescent intensity across multiple imaging rounds. **(b)** Images acquired from the ATTO 390 NHS-ester stained sample, revealing a spectral red-shift after illumination of a 405-nm excitation laser. 1st row displays a 405-nm laser exposed region, while 2nd row shows an adjacent field-of-view without illumination of the 405-nm laser. The magenta highlighted box region indicates the overlapped region between the images in the 1st row and the 2nd row. The overlapped region also exhibited a spectral red-shift due to the 405-nm laser exposure. All scale bars: 20 μm. All length scales are presented in pre-expansion dimensions.
(TIF)

S2 FigFluorophore NHS-ester screening.Each half brain slice, stained with corresponding fluorophore NHS-ester, was imaged with a 10× objective lens to confirm the labeling density and uniformity of each fluorophore NHS-ester across the entire regions of the brain slice. 488-nm excitable fluorophores are Alexa Fluor 488, CF 488A, and CF 514. 561-nm excitable fluorophores are ATTO 565, ATTO Rho 101, ATTO 594, Cy3, and CF 568. 647-nm excitable fluorophores are ATTO 633, ATTO 647N, ATTO 680, CF 660R, and CF 680R. Scale bars: 1 mm.(TIF)

S3 FigResistance to photobleaching of fluorophore NHS esters.Each half brain slice, stained with corresponding fluorophore NHS-ester, was imaged with a 60× objective lens in the same region twice, before and after 5 min illumination of excitation wavelength within 1× PBS buffer, respectively. 488-nm excitable fluorophores are Alexa Fluor 488, CF 488A, and CF 514. 561-nm excitable fluorophores are ATTO 565, ATTO Rho 101, ATTO 594, Cy3, and CF 568. 647-nm excitable fluorophores are ATTO 633, ATTO 647N, ATTO 680, CF 660R, and CF 680R. Changes in fluorescent intensity were checked by comparing the first and second images. Scale bars: 20 μm.(TIF)

S4 Fig Spectral shift of fluorophore NHS-esters. Several fluorophore NHS-esters, tested as shown in S3 Fig, experienced a spectral blue-shift within a few minutes of illumination of excitation wavelength. **(a)** Result of ATTO 594 NHS-ester with 561-nm excitation laser. ATTO 594 fluorescence signal initially had bleed-through across the 561-nm and the 647-nm detection channels. After 5 min of illumination with a 561-nm excitation laser, the fluorescence signal in the 647-nm detection channel decreased, while the signal in the 561-nm detection channel significantly increased, indicating a spectral blue-shift of the emission spectrum. **(b)** Result of ATTO 647N NHS-ester with 647-nm excitation laser. The fluorescence signal of ATTO 647N was initially visualized only within the 647-nm detection channel. After 5 min of illumination with a 647-nm excitation laser, the fluorescence signal of ATTO 647N was gradually visualized within the 561-nm detection channel, meaning a spectral blue-shift of the emission spectrum. The display conditions (minimum pixel value - maximum pixel value) were labeled on each image, and the display conditions remained consistent before and after illumination of excitation wavelength. To reduce the background noise, the minimum pixel value for all the measured images was set to 110. All scale bars: 20 μm.
(TIF)

S5 Fig Antibody compatibility test with the normal brain slices and the ATTO 565 fluorophore NHS-ester-stained brain slices. Each antibody image within both cases is displayed in identical conditions. There was no significant intensity difference between the normal and the fluorophore NHS-ester (ATTO 565 NHS-ester) stained brain slices. All scale bars: 20 μm.
(TIF)

S6 Fig Sample distortion along consecutive imaging rounds of cyclic staining without chemical de-staining. ATTO 565 NHS-ester images along five consecutive rounds of cyclic staining without chemical de-staining. Neglectable sample distortions are observed in the cyclic staining without chemical de-staining. Scale bars: Full FOV 20 μm, and cropped ROI 10 μm. All length scales are presented in pre-expansion dimensions. Number of sample *N* = 1, Number of datapoints *M* = 5 acquired from a single mouse brain slice.
(TIF)

S7 Fig Comparison of labeling density of DAPI and ATTO 680 NHS-ester in sub-regions of expanded mouse brain slice gel. **(a)** Tiled image of expanded mouse brain slice gel, stained with DAPI (blue) and ATTO 680 NHS-ester (white). **(b–d)** High-resolution image of both fiducial markers in sub-regions of expanded mouse brain slice gel (cortex, CA1, dentate gyrus and Thalamus). Scale bars: **(a)** 1 mm, **(b–d)** 50 μm and (**b–d** inset images) 5 μm. All length scales are presented in pre-expansion dimensions.
(TIF)

S8 Fig Additional data for validation of a dense label-based registration. Comparison of registration accuracy between DAPI and NHS ester was performed for six independent brain slices. Eleven z-stack images were acquired from each sample, 66 datapoints in total. The entire dataset showed that NHS-ester-based registration mostly provides more accurate registration performance. Red: anti-vGluT1 staining (1st round), Green: anti-Alexa Fluor 488 staining (2nd round). All scale bars: 5 μm. All length scales are presented in pre-expansion dimensions.
(TIF)

S9 Fig Additional quantitative analysis of registration accuracy depending on the type of registration marker. In addition to comparing the image registration accuracy between the conventional DAPI staining and fluorophore NHS-ester staining, the accuracy of image registration using the target image, vGluT1 staining, was also evaluated. Consistent with previous results, DAPI staining demonstrated relatively lower image registration accuracy. In contrast, image registration using both fluorophore NHS-ester staining and vGluT1 staining showed high levels of accuracy, with no significant difference in accuracy observed between the two resulting images. Please see S1 Data for individual numerical values of the Pearson correlation coefficient.
(TIF)

S10 Fig Validation of the absence of crosstalk in preformed antibody complexes and photobleaching-based multiplexing. **(a)** Images acquired from each imaging round and after photobleaching, confirming the robustness of the photobleaching-based multiplexing strategy and the absence of antibody crosstalk in preformed antibody complexes. **(b, c)** Resulting 3D multiplexed images acquired through photobleaching-based multiplexing. **(b)** Single channel 3D images of target proteins. **(c)** Merged 15-plex 3D image. Images acquired from each laser channel are displayed in identical brightness conditions. All scale bars: 20 μm. Number of samples *N* = 4 acquired from two independent mouse brain slices.
(TIF)

S11 Fig Zoomed-in view of S10 Fig for SOX2, GFAP, and Iba1. As reported from previous studies, SOX2-expressing cells are not positive for Iba1 and vice versa. Green highlighted boxes show that GFAP expression is highly colocalized with SOX2 positive cells but not for Iba1 positive cells. Left column: Maximum intensity projection (MIP) view, right column: Blend rendering view. All scale bars: 20 μm.
(TIF)

S12 Fig Quantitative validation of the accuracy of signal unmixing. Pearson correlation coefficients were measured to assess the accuracy of signal unmixing by comparing unmixed images and their corresponding ground-truth images. A total of 27 datapoints were obtained from two different sample types: 21 from 10-μm-thick cryo-sectioned brain slices and 6 from 150-μm-thick brain slices. Images were acquired from various sub-regions of mouse brain slices, including the CA, cortex, and dentate gyrus. The average Pearson correlation coefficient was approximately 0.98, indicating a strong agreement match with the ground-truth images. Among the 27 data points, 21 were measured between the first and second rounds, while 6 were measured between the second and third rounds. Please see S2 Data for individual numerical values of the Pearson correlation coefficient.
(TIF)

S13 Fig Registration of multiplexed images *via* Cy3 NHS-ester staining in a mouse brain slice. **(a)** Composite image generated by registering three images acquired in separate staining and imaging rounds. Imager DNA labeled with Alexa Fluor 488 was used in all three rounds. DAPI-labeled nuclei were used as fiducial markers. Green, the first round (MAP2 and Bassoon); red, the second round (GFAP and Bassoon); blue, the third round (GFAP and Homer1). **(b)** Magnified view of the boxed region in **a**. **(c, d)** Line profiles (dots) of the GFAP of the second-round image (red) and the third-round image (blue) with a superimposed fit with the Gaussian (solid lines) along the dotted lines in **b**. **(e)** As in **a**, structures labeled with Cy3 NHS-ester were used as fiducial markers (the Cy3 channel is not shown). **(f)** Magnified view of the boxed region in **e**. **(g, h)** Line profiles (dots) of Bassoon of the first-round image (green) and the second-round image (red), with a superimposed fit with Gaussian (solid lines) along the dotted lines in **f**. **(i–l)** Cropped sub-ROIs of Bassoon and GFAP structures registered by DAPI and NHS-ester, respectively. **(m)** Pearson correlation coefficient data plot for Bassoon and GFAP images. Please see S3 Data for individual numerical values of the line profiling plots and S4 Data for individual numerical values of the Pearson correlation coefficient. Scale bars: **(a)** 10 µm; **(b)** 500 nm; **(c)** 10 µm; **(d)** 500 nm. All length scales are presented in pre-expansion dimensions. Number of sample *N* = 1, Number of sub-ROIs = 4 acquired from a single mouse brain slice.
(TIF)

S14 Fig Sequential imaging in expanded cultured cells with actin as a registration marker. **(a, b)** Single-channel images of phalloidin-labeled actin fibers in each imaging round: **a**, 1st round; **b**, 2nd round. **(c)** Image from the 1st round. Gray, DAPI; red, Actin; green, Vimentin and Lamin A/C; blue, CCP and Cytokeratin 8/18. **(d)** Image from the 2nd round. Gray, DAPI; red, Actin; green, Vimentin and CCP; blue, Lamin A/C and Cytokeratin 8/18. Yellow boxes display 6-color images, including the DAPI channel. Scale bars: **(a–d)** 5 μm; insets of **c** and **d**, 2 μm. All length scales are presented in pre-expansion dimensions. Number of sample *N* = 1 acquired from a single substrate.
(TIF)

S15 Fig Signal amplification *via* in situ silver growth in Alexa 647—FluoroNanogold stained microtubule within cells. **(a)** Pre-silver growth image of β-tubulin labeled BS-C-1 cell. **(b)** Post-silver growth image of the same cell in **a**. **(c)** Fluorescence intensity comparison between pre- and post-silver growth (*n* = 4, Avg (±STD), pre: 216.28 (±37.08), post: 264.94 (±63.78)). Scale bars: 30 μm. Number of sample *N* = 4 acquired from a single substrate.
(TIF)

S1 Table Quantification of photobleaching resistance of fluorophore NHS-esters in S2 Fig. Intensity drop rate before and after 5 min-excitation laser illumination with identical imaging conditions was measured. 488-nm excitable fluorophore NHS-esters experienced significant photobleaching, providing about an 80% intensity drop rate. ATTO 594 and ATTO 647N showed spectral blue shift under 5 min-illumination. ATTO 594 initially has some degree of bleed-through across 561- and 647-nm imaging channels and showed enhanced fluorescence intensity in 561-nm imaging channel after 5 min-illumination, inferring the spectral blue shift of emission spectrum.
(XLSX)

S2 Table List of reagents and supplies used in this study. (XLSX)

S3 Table List of labeling reagents and their final concentration for use. (XLSX)

S4 Table List of DNA oligos used for manual conjugation. (XLSX)

S5 Table List of imager DNAs for sequential imaging. (XLSX)

S1 Data Quantitative analysis of image registration accuracy between sparse- and dense-labeled markers for Figs 2, S8, and S9 (Measurement of Pearson correlation coefficient). (XLSX)

S2 Data Quantitative analysis of signal unmixing accuracy within thick brain slices and cryo-sectioned brain slices for S12 Fig (Measurement of Pearson correlation coefficient). (XLSX)

S3 Data Quantitative analysis of image registration accuracy between sparse- and dense-labeled markers for S13c, S13d, S13g and S13h Fig (Measurement of peak-to-peak distance by line profiling). (XLSX)

S4 Data Quantitative analysis of image registration accuracy between sparse- and dense labeled markers for S13m Fig (Measurement of Pearson correlation coefficient). (XLSX)

## Abbreviations

DHEBA: *N,N*-(1,2-dihydroxyethylene) bisacrylamide
DMSO: dimethyl sulfoxide
eMAP: epitope-preserving magnified analysis of the proteome
ExM: Expansion Microscopy
FOV: field-of-view
GFAP: glial fibrillary acidic protein
Iba1: ionized calcium-binding adapter molecule 1
MAP: Magnified Analysis of Proteome
MBP: Myelin basic protein
NDS: normal donkey serum
NHS: *N*-hydroxysuccinimide
PBS: phosphate-buffered saline
PCC: Pearson correlation coefficient
RT: room temperature
SDS: sodium dodecyl sulfate
SOX2: SRY-box 2
3D: three-dimensional
