# Supplementary figures and images for: Highly accurate image registration for 3D multiplexed cyclic imaging using dense labeling in expandable tissue gels
Source: PLoS Biol. 2025 Jul 3;23(7):e3003240. doi: 10.1371/journal.pbio.3003240 (PMC12225881; doi:10.1371/journal.pbio.3003240)

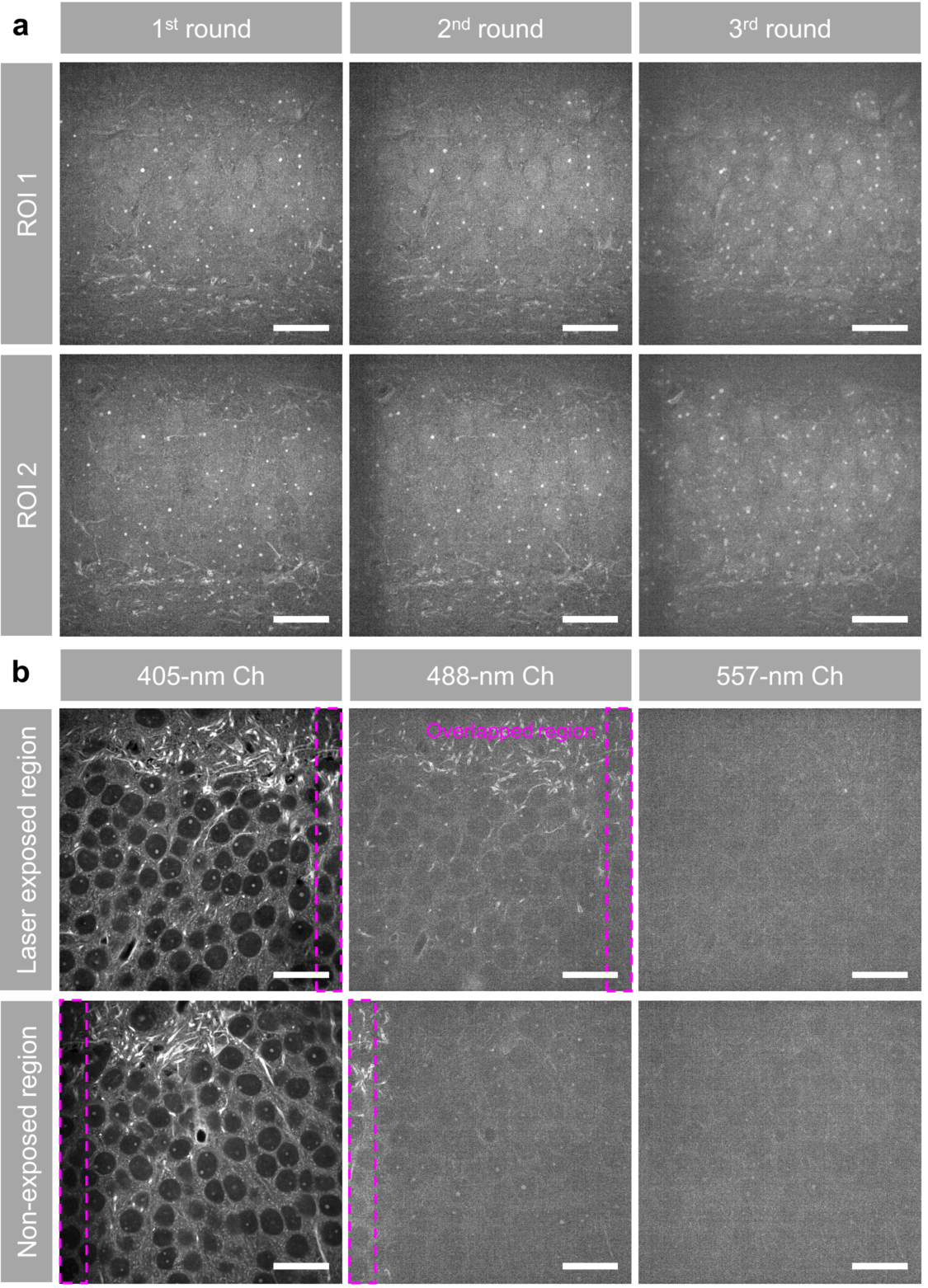

Supplement: S1 Fig — (a) Images acquired from the CF 405S NHS-ester stained sample, showing low fluorescent intensity across multiple imaging rounds. (b) Images acquired from the ATTO 390 NHS-ester stained sample, revealing a spectral red-shift after illumination of a 405-nm excitation laser. 1st row displays a 405-nm laser exposed region, while 2nd row shows an adjacent field-of-view without illumination of the 405-nm laser. The magenta highlighted box region indicates the overlapped region between the images in the 1st row and the 2nd row. The overlapped region also exhibited a spectral red-shift due to the 405-nm laser exposure. All scale bars: 20 μm. All length scales are presented in pre-expansion dimensions. (TIF) [file pbio.3003240.s001.tif]

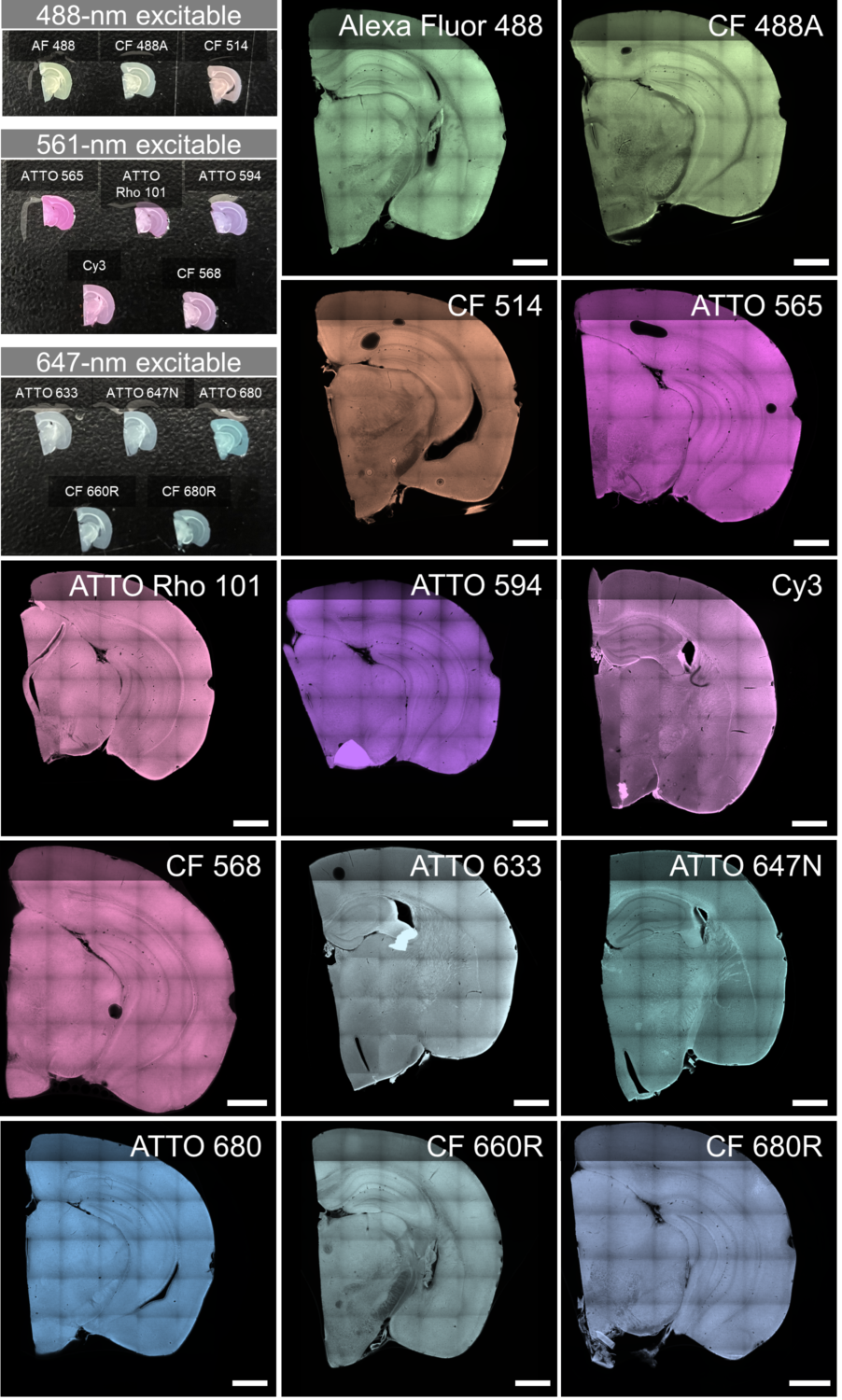

Supplement: S2 Fig — Each half brain slice, stained with corresponding fluorophore NHS-ester, was imaged with a 10× objective lens to confirm the labeling density and uniformity of each fluorophore NHS-ester across the entire regions of the brain slice. 488-nm excitable fluorophores are Alexa Fluor 488, CF 488A, and CF 514. 561-nm excitable fluorophores are ATTO 565, ATTO Rho 101, ATTO 594, Cy3, and CF 568. 647-nm excitable fluorophores are ATTO 633, ATTO 647N, ATTO 680, CF 660R, and CF 680R. Scale bars: 1 mm. (TIF) [file pbio.3003240.s002.tif]

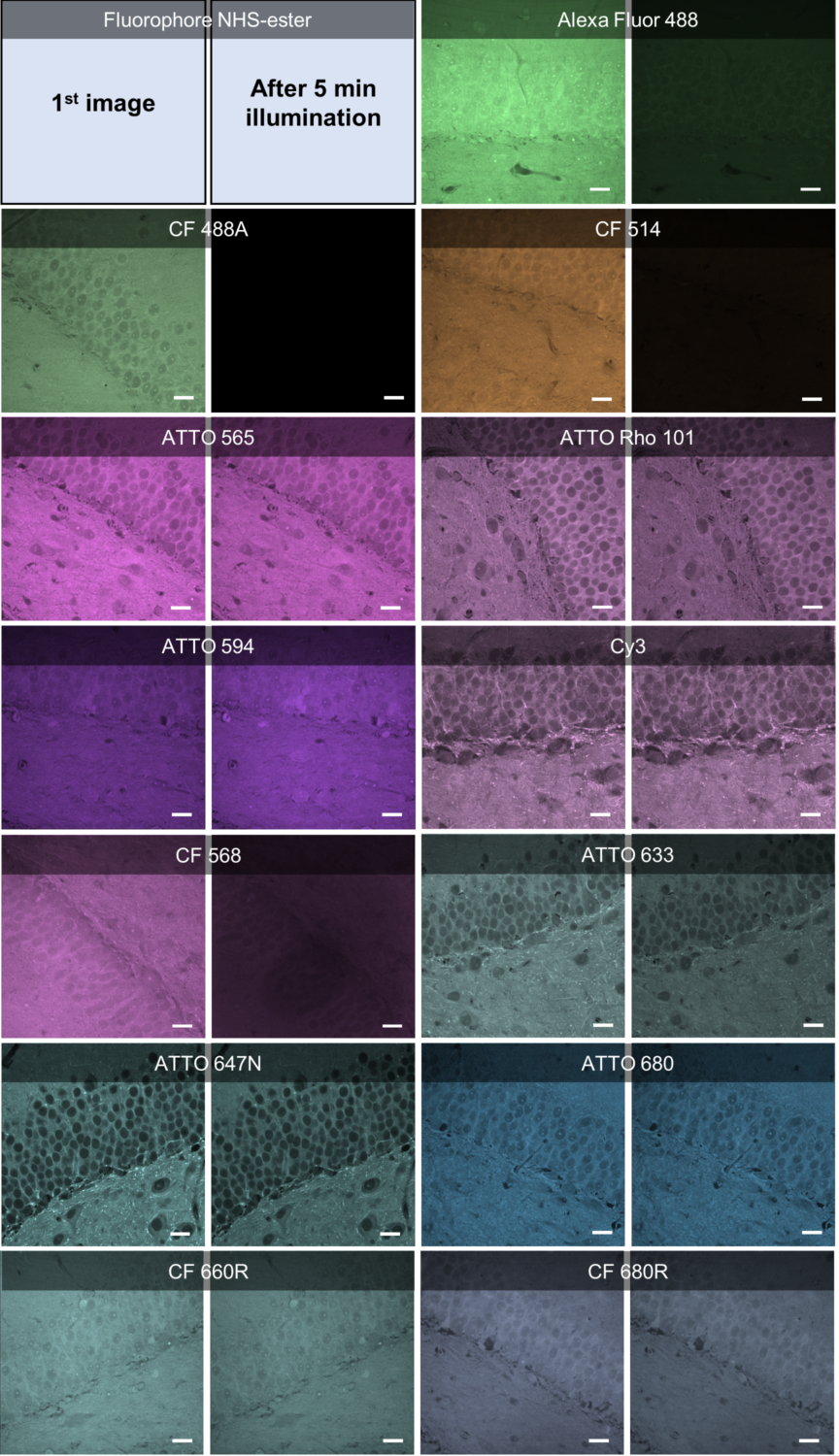

Supplement: S3 Fig — Each half brain slice, stained with corresponding fluorophore NHS-ester, was imaged with a 60× objective lens in the same region twice, before and after 5 min illumination of excitation wavelength within 1× PBS buffer, respectively. 488-nm excitable fluorophores are Alexa Fluor 488, CF 488A, and CF 514. 561-nm excitable fluorophores are ATTO 565, ATTO Rho 101, ATTO 594, Cy3, and CF 568. 647-nm excitable fluorophores are ATTO 633, ATTO 647N, ATTO 680, CF 660R, and CF 680R. Changes in fluorescent intensity were checked by comparing the first and second images. Scale bars: 20 μm. (TIF) [file pbio.3003240.s003.tif]

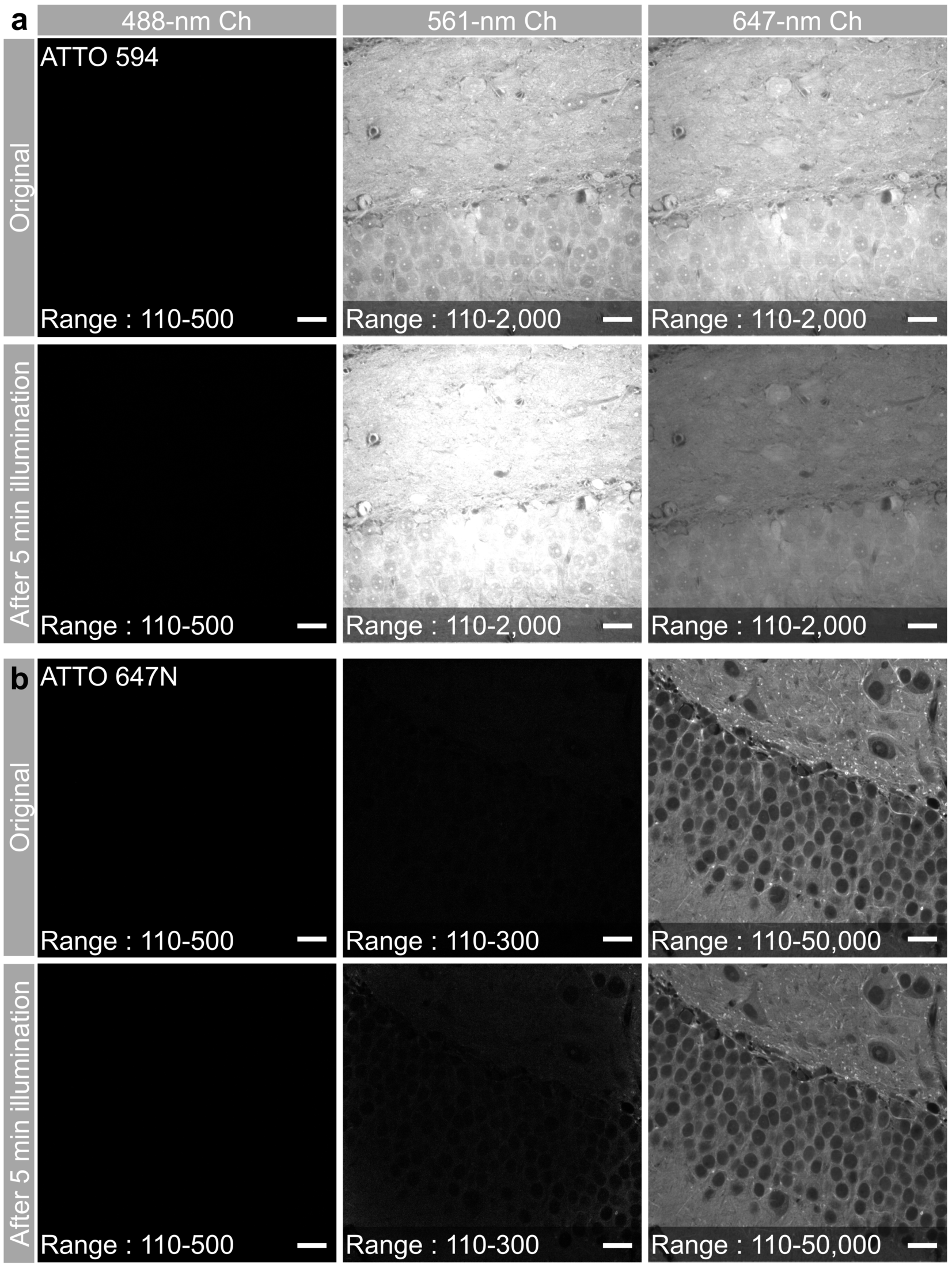

Supplement: S4 Fig — Several fluorophore NHS-esters, tested as shown in S3 Fig, experienced a spectral blue-shift within a few minutes of illumination of excitation wavelength. (a) Result of ATTO 594 NHS-ester with 561-nm excitation laser. ATTO 594 fluorescence signal initially had bleed-through across the 561-nm and the 647-nm detection channels. After 5 min of illumination with a 561-nm excitation laser, the fluorescence signal in the 647-nm detection channel decreased, while the signal in the 561-nm detection channel significantly increased, indicating a spectral blue-shift of the emission spectrum. (b) Result of ATTO 647N NHS-ester with 647-nm excitation laser. The fluorescence signal of ATTO 647N was initially visualized only within the 647-nm detection channel. After 5 min of illumination with a 647-nm excitation laser, the fluorescence signal of ATTO 647N was gradually visualized within the 561-nm detection channel, meaning a spectral blue-shift of the emission spectrum. The display conditions (minimum pixel value - maximum pixel value) were labeled on each image, and the display conditions remained consistent before and after illumination of excitation wavelength. To reduce the background noise, the minimum pixel value for all the measured images was set to 110. All scale bars: 20 μm. (TIF) [file pbio.3003240.s004.tif]

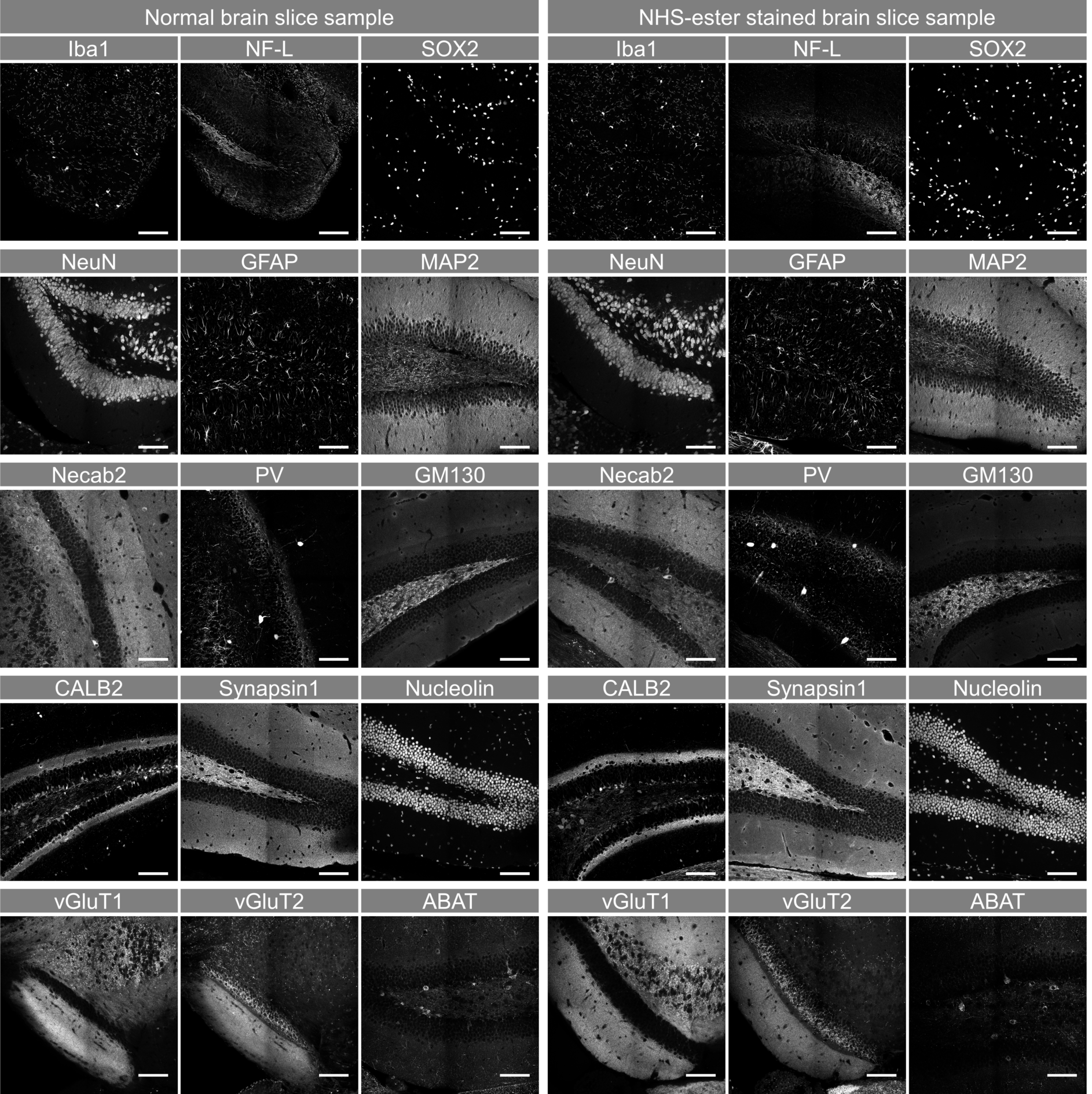

Supplement: S5 Fig — Each antibody image within both cases is displayed in identical conditions. There was no significant intensity difference between the normal and the fluorophore NHS-ester (ATTO 565 NHS-ester) stained brain slices. All scale bars: 20 μm. (TIF) [file pbio.3003240.s005.tif]

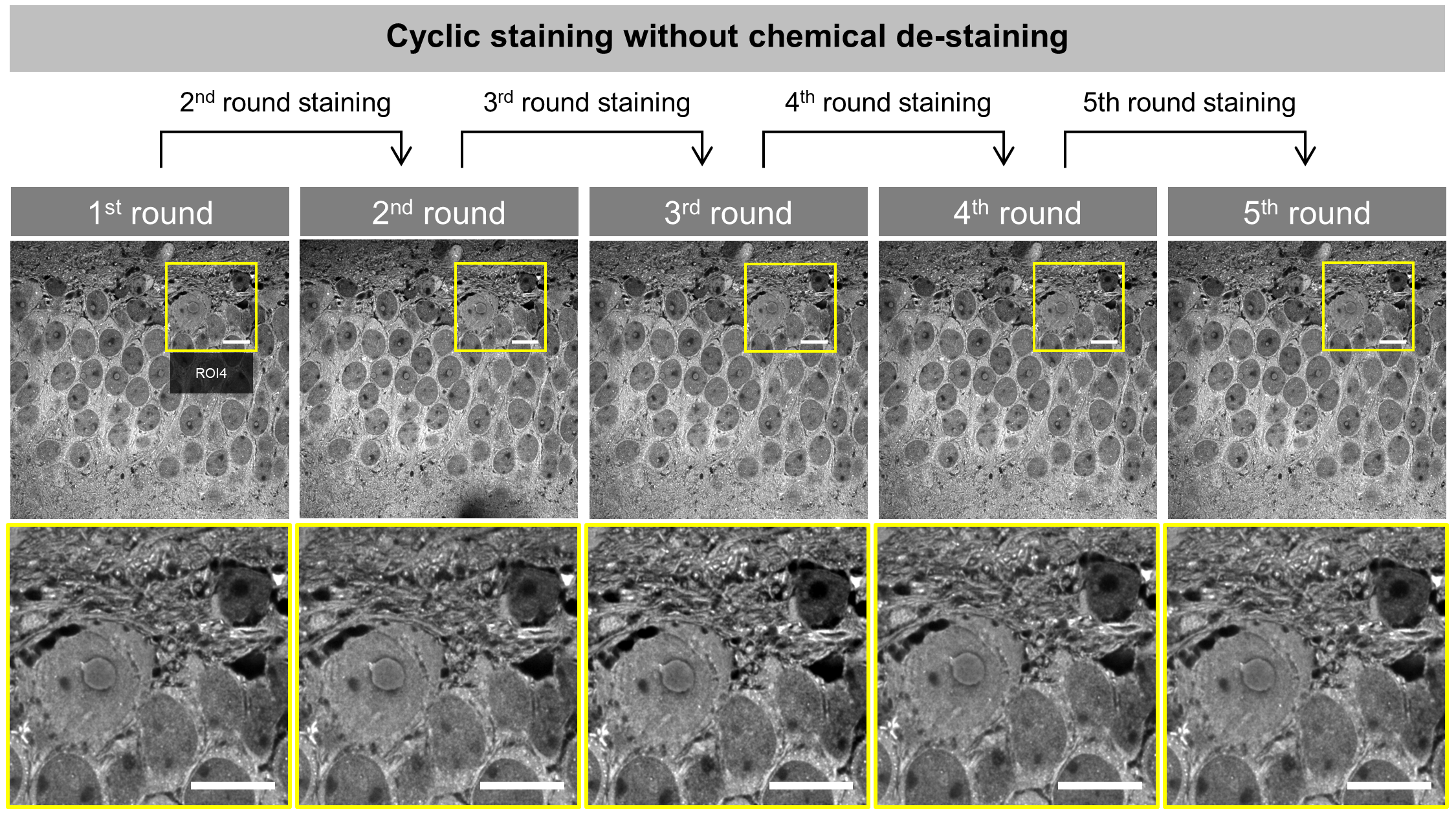

Supplement: S6 Fig — ATTO 565 NHS-ester images along five consecutive rounds of cyclic staining without chemical de-staining. Neglectable sample distortions are observed in the cyclic staining without chemical de-staining. Scale bars: Full FOV 20 μm, and cropped ROI 10 μm. All length scales are presented in pre-expansion dimensions. Number of sample N = 1, Number of datapoints M = 5 acquired from a single mouse brain slice. (TIF) [file pbio.3003240.s006.TIF]

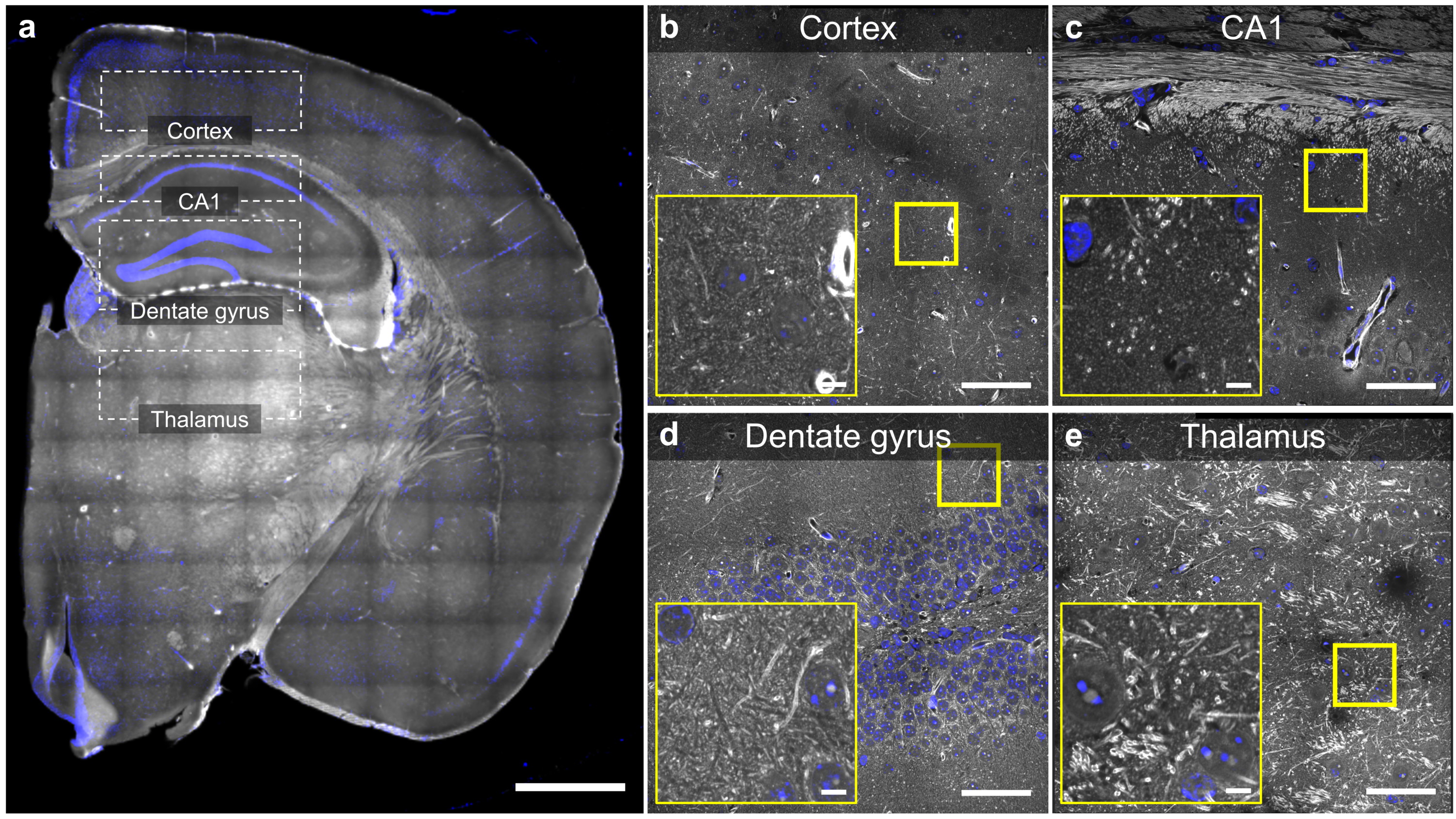

Supplement: S7 Fig — (a) Tiled image of expanded mouse brain slice gel, stained with DAPI (blue) and ATTO 680 NHS-ester (white). (b–d) High-resolution image of both fiducial markers in sub-regions of expanded mouse brain slice gel (cortex, CA1, dentate gyrus and Thalamus). Scale bars: (a) 1 mm, (b–d) 50 μm and (b–d inset images) 5 μm. All length scales are presented in pre-expansion dimensions. (TIF) [file pbio.3003240.s007.tif]

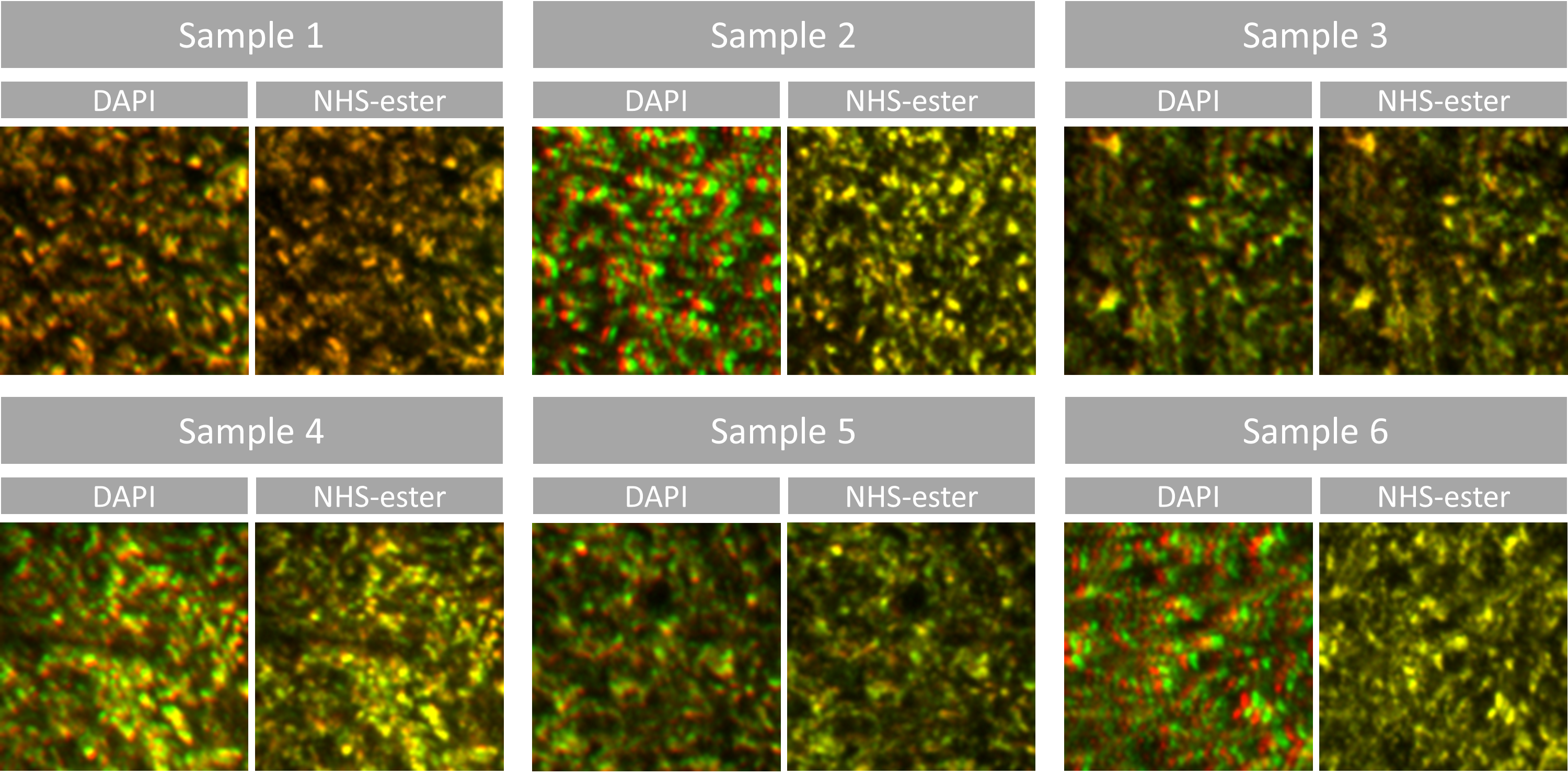

Supplement: S8 Fig — Comparison of registration accuracy between DAPI and NHS ester was performed for six independent brain slices. Eleven z-stack images were acquired from each sample, 66 datapoints in total. The entire dataset showed that NHS-ester-based registration mostly provides more accurate registration performance. Red: anti-vGluT1 staining (1st round), Green: anti-Alexa Fluor 488 staining (2nd round). All scale bars: 5 μm. All length scales are presented in pre-expansion dimensions. (TIF) [file pbio.3003240.s008.TIF]

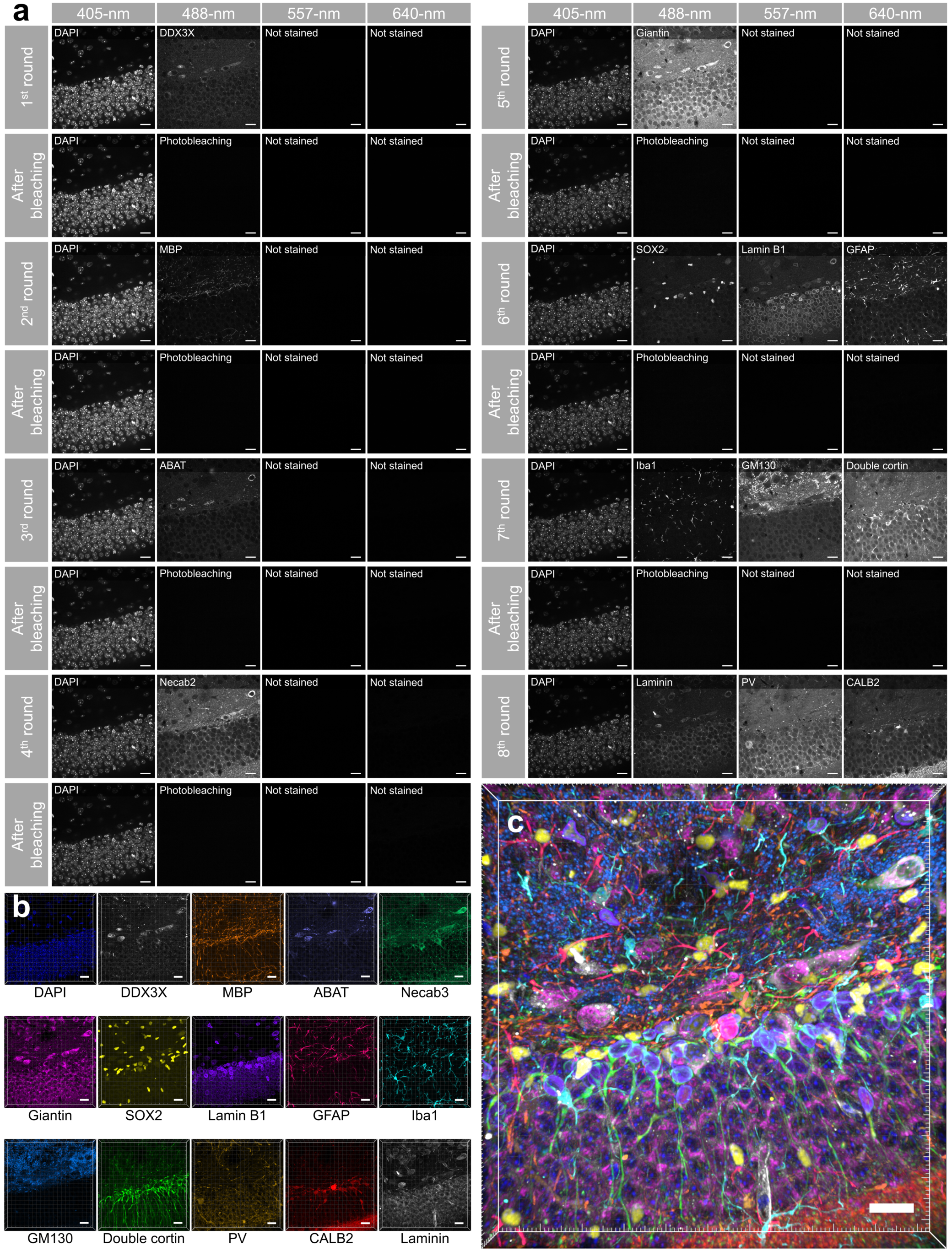

Supplement: S10 Fig — (a) Images acquired from each imaging round and after photobleaching, confirming the robustness of the photobleaching-based multiplexing strategy and the absence of antibody crosstalk in preformed antibody complexes. (b, c) Resulting 3D multiplexed images acquired through photobleaching-based multiplexing. (b) Single channel 3D images of target proteins. (c) Merged 15-plex 3D image. Images acquired from each laser channel are displayed in identical brightness conditions. All scale bars: 20 μm. Number of samples N = 4 acquired from two independent mouse brain slices. (TIF) [file pbio.3003240.s010.tif]

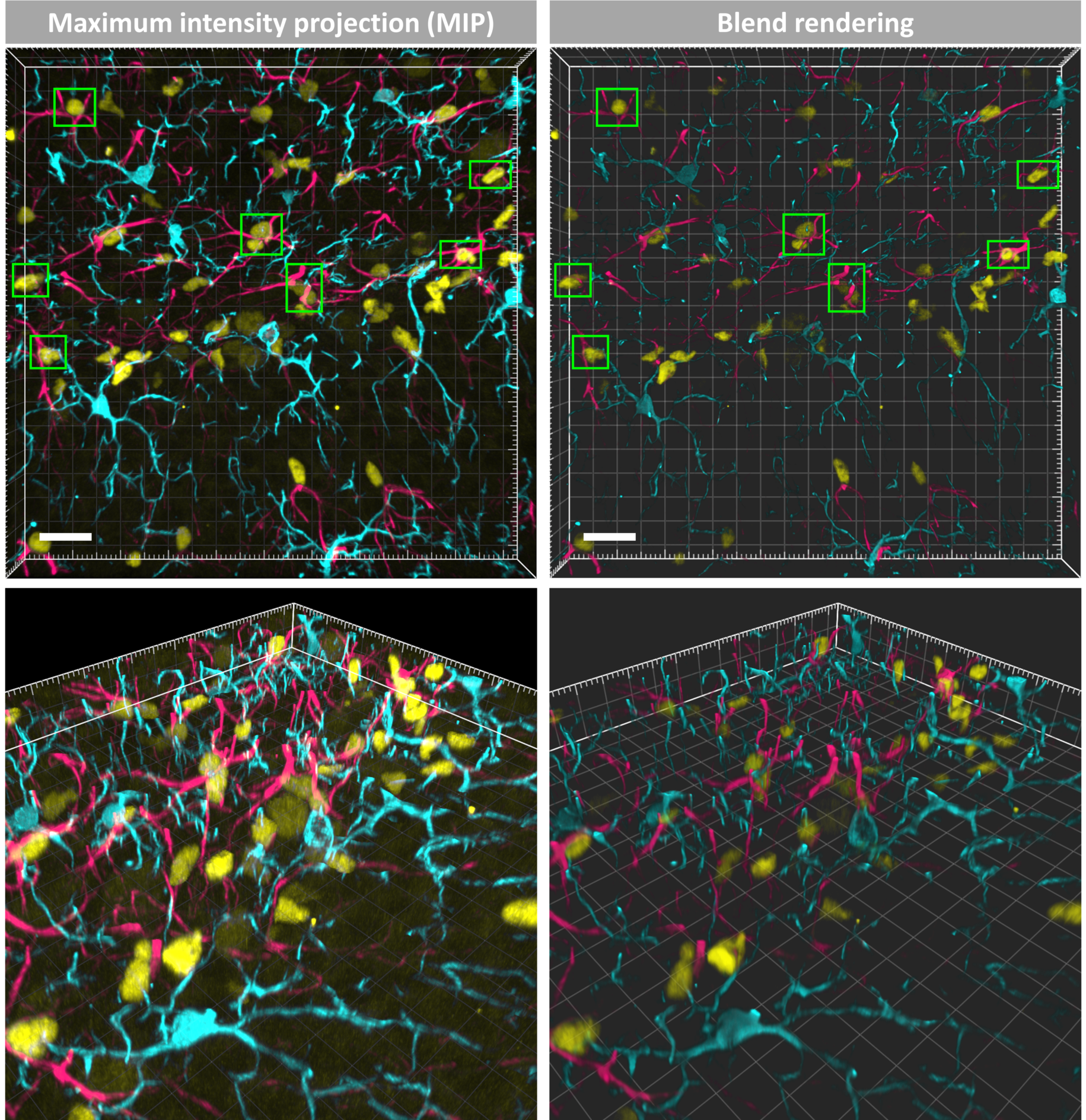

Supplement: S11 Fig — As reported from previous studies, SOX2-expressing cells are not positive for Iba1 and vice versa. Green highlighted boxes show that GFAP expression is highly colocalized with SOX2 positive cells but not for Iba1 positive cells. Left column: Maximum intensity projection (MIP) view, right column: Blend rendering view. All scale bars: 20 μm. (TIF) [file pbio.3003240.s011.tif]

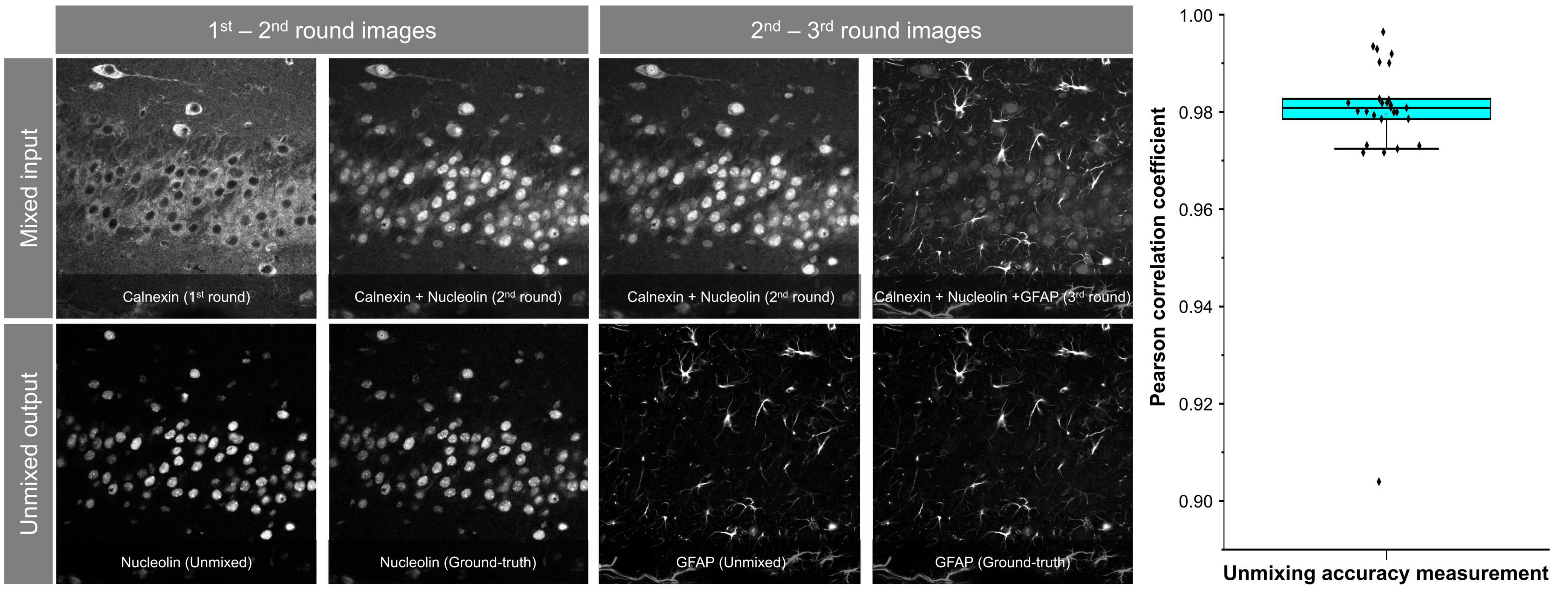

Supplement: S12 Fig — Pearson correlation coefficients were measured to assess the accuracy of signal unmixing by comparing unmixed images and their corresponding ground-truth images. A total of 27 datapoints were obtained from two different sample types: 21 from 10-μm-thick cryo-sectioned brain slices and 6 from 150-μm-thick brain slices. Images were acquired from various sub-regions of mouse brain slices, including the CA, cortex, and dentate gyrus. The average Pearson correlation coefficient was approximately 0.98, indicating a strong agreement match with the ground-truth images. Among the 27 data points, 21 were measured between the first and second rounds, while 6 were measured between the second and third rounds. Please see S2 Data for individual numerical values of the Pearson correlation coefficient. (TIF) [file pbio.3003240.s012.tif]

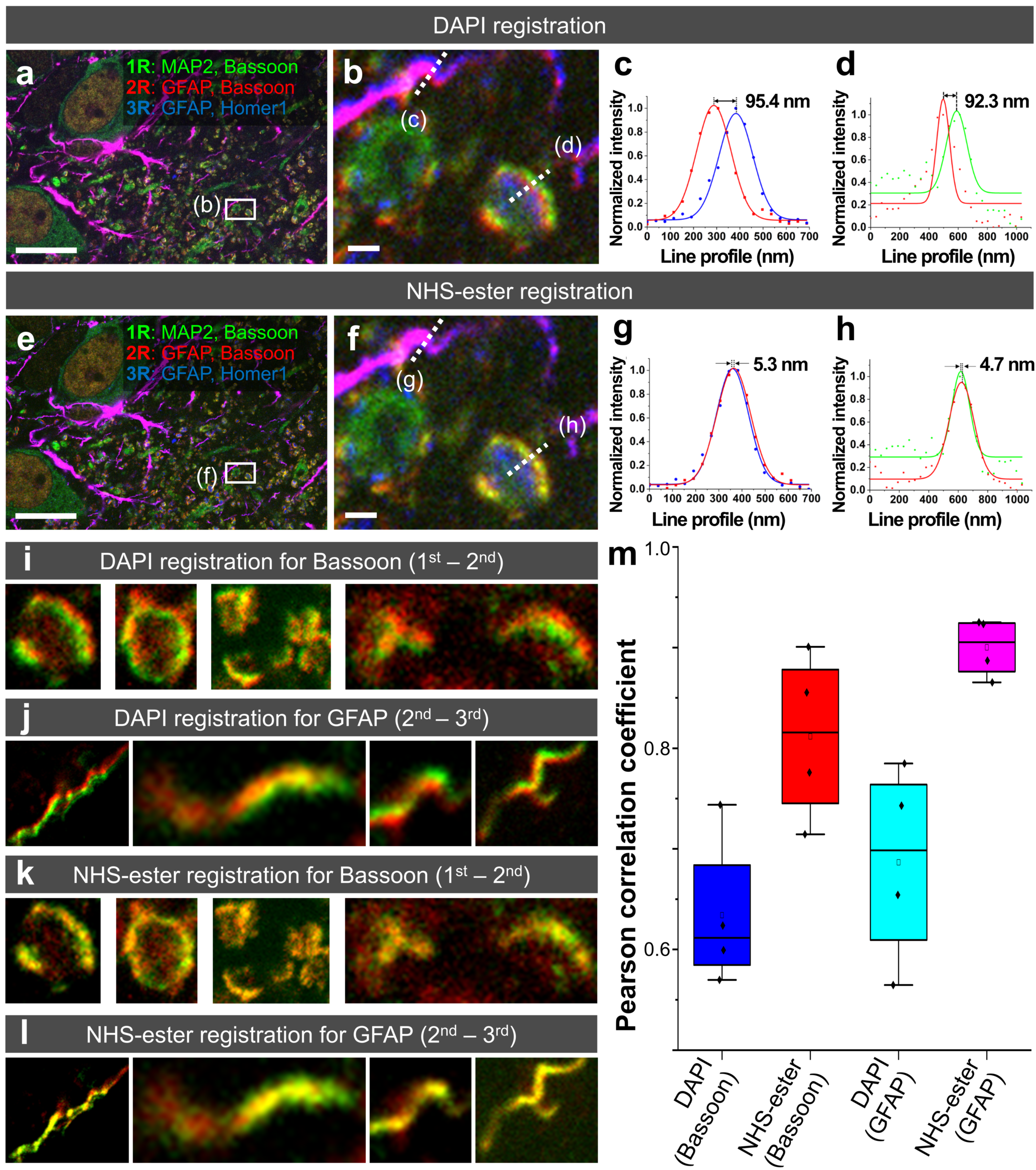

Supplement: S13 Fig — (a) Composite image generated by registering three images acquired in separate staining and imaging rounds. Imager DNA labeled with Alexa Fluor 488 was used in all three rounds. DAPI-labeled nuclei were used as fiducial markers. Green, the first round (MAP2 and Bassoon); red, the second round (GFAP and Bassoon); blue, the third round (GFAP and Homer1). (b) Magnified view of the boxed region in a. (c, d) Line profiles (dots) of the GFAP of the second-round image (red) and the third-round image (blue) with a superimposed fit with the Gaussian (solid lines) along the dotted lines in b. (e) As in a, structures labeled with Cy3 NHS-ester were used as fiducial markers (the Cy3 channel is not shown). (f) Magnified view of the boxed region in e. (g, h) Line profiles (dots) of Bassoon of the first-round image (green) and the second-round image (red), with a superimposed fit with Gaussian (solid lines) along the dotted lines in f. (i–l) Cropped sub-ROIs of Bassoon and GFAP structures registered by DAPI and NHS-ester, respectively. (m) Pearson correlation coefficient data plot for Bassoon and GFAP images. Please see S3 Data for individual numerical values of the line profiling plots and S4 Data for individual numerical values of the Pearson correlation coefficient. Scale bars: (a) 10 µm; (b) 500 nm; (c) 10 µm; (d) 500 nm. All length scales are presented in pre-expansion dimensions. Number of sample N = 1, Number of sub-ROIs = 4 acquired from a single mouse brain slice. (TIF) [file pbio.3003240.s013.tif]

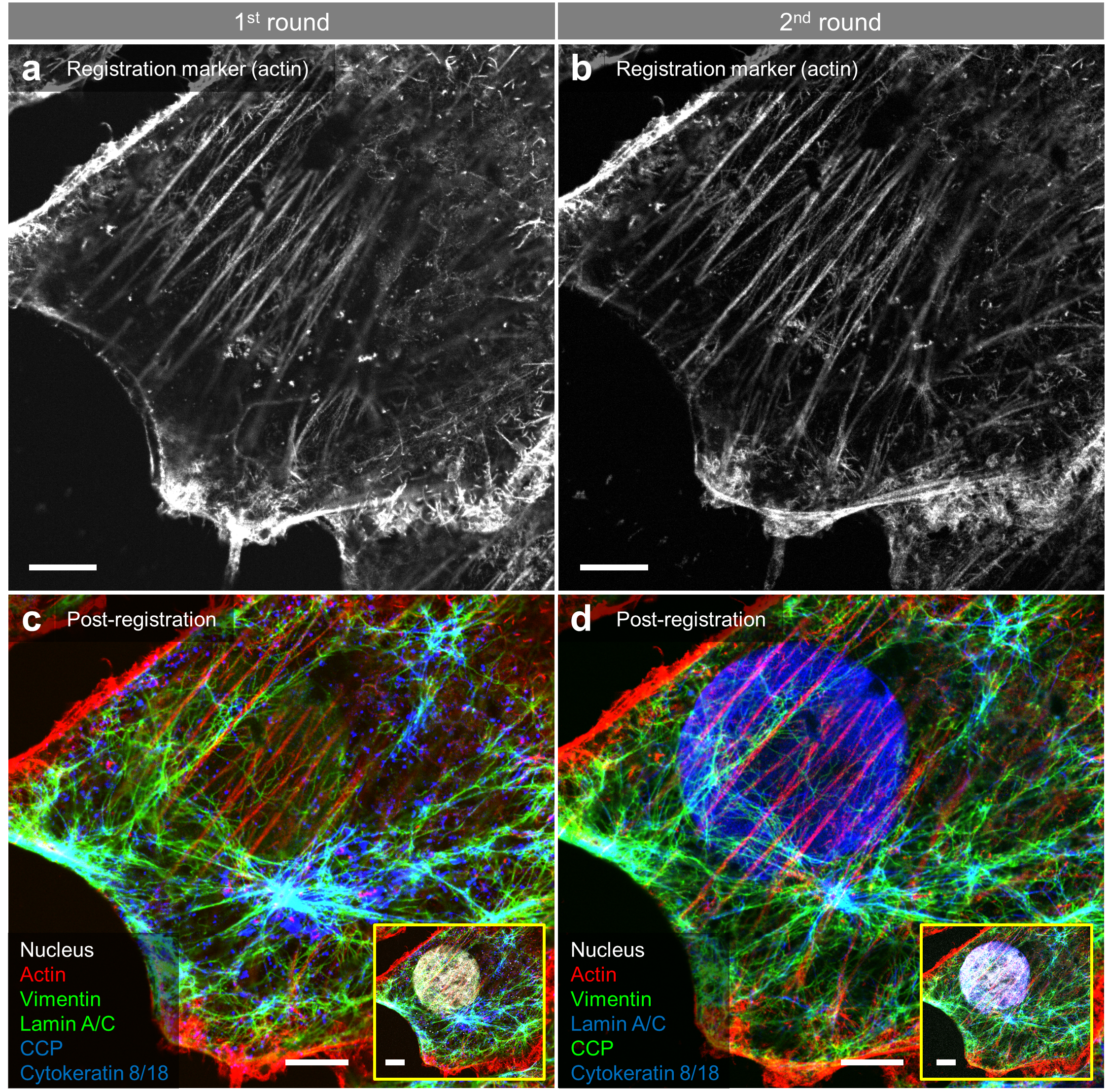

Supplement: S14 Fig — (a, b) Single-channel images of phalloidin-labeled actin fibers in each imaging round: a, 1st round; b, 2nd round. (c) Image from the 1st round. Gray, DAPI; red, Actin; green, Vimentin and Lamin A/C; blue, CCP and Cytokeratin 8/18. (d) Image from the 2nd round. Gray, DAPI; red, Actin; green, Vimentin and CCP; blue, Lamin A/C and Cytokeratin 8/18. Yellow boxes display 6-color images, including the DAPI channel. Scale bars: (a–d) 5 μm; insets of c and d, 2 μm. All length scales are presented in pre-expansion dimensions. Number of sample N = 1 acquired from a single substrate. (TIF) [file pbio.3003240.s014.TIF]

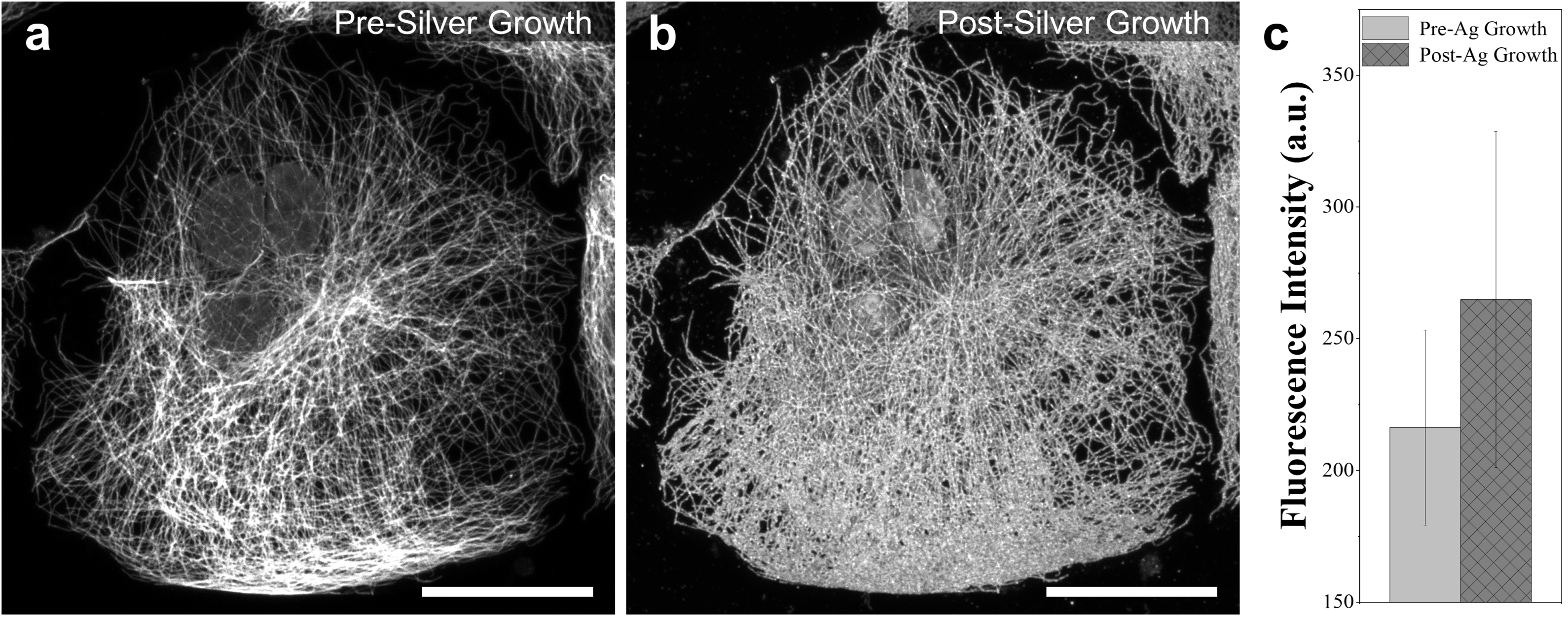

Supplement: S15 Fig — (a) Pre-silver growth image of β-tubulin labeled BS-C-1 cell. (b) Post-silver growth image of the same cell in a. (c) Fluorescence intensity comparison between pre- and post-silver growth (n = 4, Avg (±STD), pre: 216.28 (±37.08), post: 264.94 (±63.78)). Scale bars: 30 μm. Number of sample N = 4 acquired from a single substrate. (TIF) [file pbio.3003240.s015.tif]
